# Ediacaran-Cambrian paleosols of Nevada and California

**DOI:** 10.1371/journal.pone.0325547

**Published:** 2025-06-24

**Authors:** Gregory J. Retallack

**Affiliations:** 1 Department of Earth Sciences, University of Oregon, Eugene, Oregon, United States of America; Birbal Sahni Institute of Palaeosciences: Birbal Sahni Institute of Palaeobotany, INDIA

## Abstract

The Cambrian and Ediacaran sequence of California and Nevada is rife with unconformities, paleovalleys, paleosols, and fluvial facies. This study confirms shallow marine environments for grey stromatolitic dolostone and shale of northern localities (Mt Dunfee and Westgard Pass), but fluvial red sandstones and siltstone of southern localities (Johnnie, Eagle Peak, Emigrant Pass, Donna Loy, and Cadiz) include paleosols as evidence for coastal plain and fluvial environments. Three marine transgressions into the southern localities, were in Ediacaran Johnnie Formation, earliest Cambrian *Manykodes pedum* zone, and Early Cambrian *Olenellus* trilobite zone. The southern locations have paleosols with Ediacaran fossils *Ernietta*, *Pteridinium*, *Swartpuntia*, and *Hallidaya* in growth position, as evidence that these vendobiont fossils were non marine. The paleosols include aridland Gypsids and Calcids, as well as weakly developed soils, with diagnostic LYREE enrichment, and low boron content of paleosols. Northern Ediacaran marine rocks, in contrast, are limestones with *Cloudina* and *Wyattia*, and shales with *Conotubus* and *Wutubus*. Identical marine and non-marine facies and biotas are also known from Ediacaran and Cambrian rocks of Namibia. Ediacaran marine wormlike fossils (Wormworld) were ecologically distinct and geographically separated from non-marine, sessile, vendobionts (Mattressland).

## Introduction

Ediacaran-Cambrian successions of the southern Great Basin in California and Nevada have been used as evidence for the Cambrian evolutionary explosion of marine life [1–3] and marine substrate revolution due to increased diversity and depth of bioturbation [4–6]. However, the sequence has long been known to be rife with disconformities and paleosols [7–8]. The best known paleosol is the sub-Cambrian “Great Unconformity” [9–11]. Other breaks in marine deposition are represented by fluvial facies and paleovalleys at several stratigraphic levels in the Stirling Quartzite and Wood Canyon Formation [12–19]. This study describes non-marine facies and paleosols, and what they can reveal about life and paleoclimate on land during the Ediacaran-Cambrian transition.

Ediacaran paleosols have been controversial [20–22], because they include fossils traditionally assumed to have been marine [23–24]. A part of the problem is that Precambrian paleosols lack diagnostic root traces of vascular plants distinguishing Silurian and younger paleosols [25–26]. But Ediacaran paleosols show differentiated soil horizons below a truncated land surface, and soil structures, such as desert pavement, ferrans, calcareous nodules, and gypsum desert roses [21,27,28]. The primary aim of this study was to examine environmental and biotic changes on land through the Ediacaran-Cambrian transition in southern California. In order to discriminate marine from non-marine Ediacaran and Cambrian rocks, this study marshalls comprehensive quantitative petrographic and geochemical data, as well as evidence from geochemical mass balance [29], boron assay [30], stable isotopic correlation [31], and YREE analysis [32]. New observations of these kinds are described and then interpreted here.

A second aim of this study is comparison of paleosols and facies with those of Namibia, because Nevada and California Ediacaran fossils *Cloudina*, *Ernietta*, *Pteridinium* and *Swartpuntia* [33–37] are identical to those of Namibia [38–41]. The Ediacaran-Cambrian marine evolutionary explosion and substrate revolution have been studied in Namibian rocks [42–44], and there are also Namibian Ediacaran paleosols [30,45].

## Geological background

**Fig 1:**
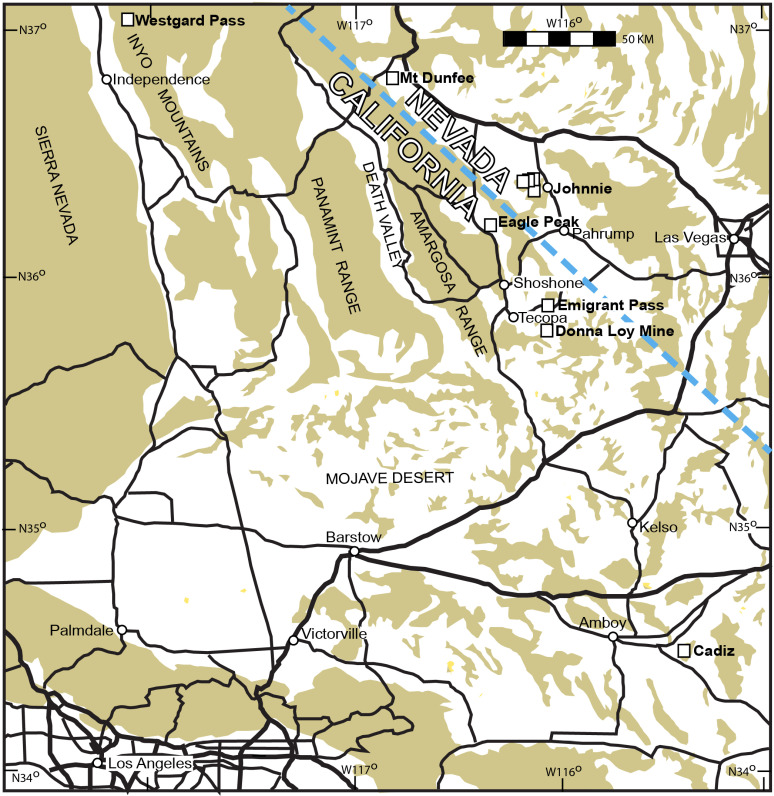
Examined localities in southern California and Nevada, USA.

**Fig 2:**
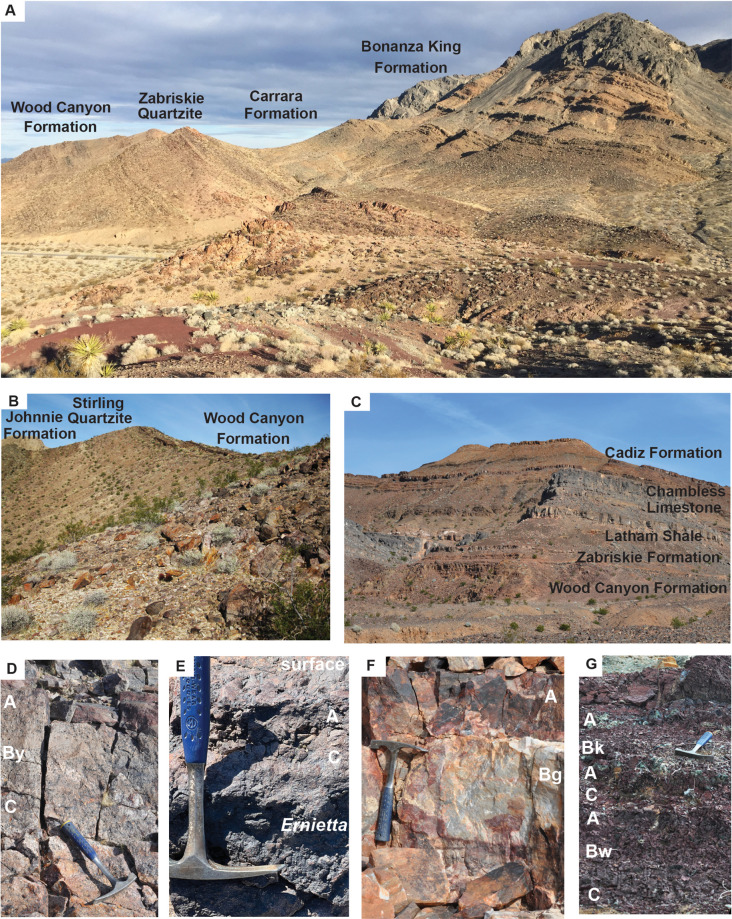
Field photographs of (A) Cambrian sequence around red bed paleosols of the Carrara Formation in Emigrant Pass; (B) late Ediacaran sequence near Donna Loy Mine, California; (C) Cambrian sequence near Cadiz, California; (D) Hebinga loam paleosol in Stirling Quartzite near Donna Loy Mine (45 m in Fig 3A); (E) Duhubite loam paleosol with surface desert pavement in Lower Member of Wood Canyon Formation west of Johnnie, Nevada; (F) Buinga sandy loam in Zabriskie Quartzite near Cadiz (19 m in Fig 3C); (G) red bed paleosols (Bui silty clay loam, Angebite silty clay loam, Pohonta silty clay loam, on Wookki silty clay loam) in Carrara Formation in Emigrant Pass (74 m in Fig 3B). Notations in white (D-G) are soil horizon interpretations based on petrographic and geochemical data (Figs 6-7).

Ediacaran and Cambrian rocks are well exposed in deserts of southern California and Nevada (Figs. 1–2). Ediacaran fossils of California are of two divergent assemblages which are found in very distinct sedimentary facies; 1, grey to green limestones and shales with stromatolites, and tubular fossils (*Cloudina*, *Conotubus*, *Wutubus*) [46–49], mostly to the north in the Inyo Mountains to Mount Dunfee, and 2, red to brown sandstones with vendobiont fossils (*Ernietta, Pteridinium*, and *Swartpuntia*) [33,50], mostly to the south near Pahrump and the Mojave Desert (Fig 1). The tubular fossils in grey marine shale and limestone have been nicknamed Ediacaran Wormworld [51]. The red sandstone assemblage dubbed Ediacaran Mattressland [30] has been variously interpreted as non-marine [21] or marine [23].

**Fig 3:**
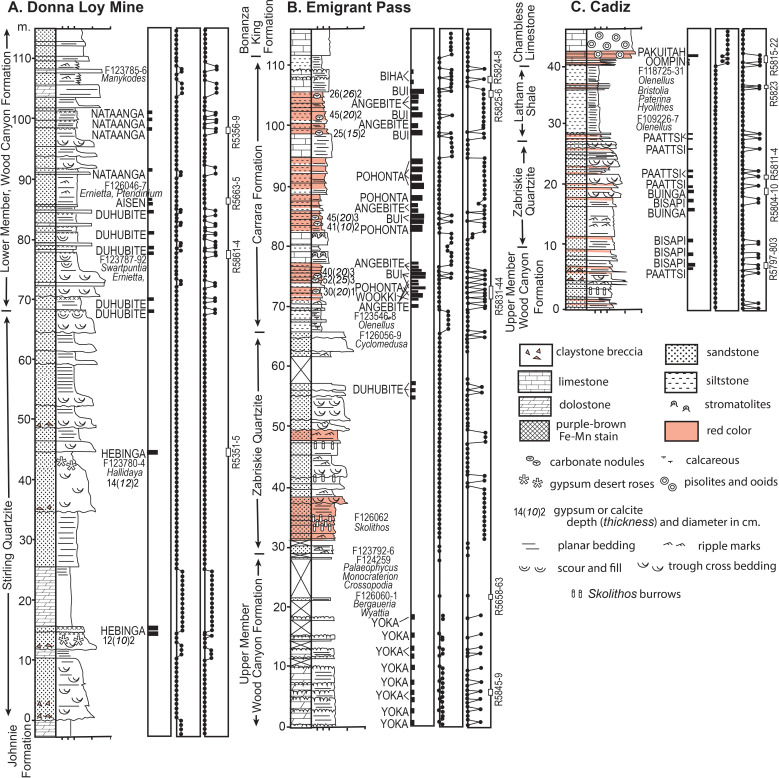
Measured sections at Donna Loy Mine (A), Emigrant Pass (B), and Cadiz (C) showing lithologies and stratigraphic levels of paleosols. Development and calcareousness by reaction with dilute HCl are from scale of Retallack (2012). Munsell Hue is from Munsell Color Company.

The base of the Cambrian is recognized by the first appearance of the marine trace fossil *Manykodes pedum* [2,52] within the upper Lower Member of the Wood Canyon Formation (Fig 1A), as well as small shelly fossils [1,46]. *Manykodes pedum* is sometimes referred to the ichnogenus *Treptichnus* [53], but those Pennsylvanian and younger insect larval traces have straight segments never seen in *M. pedum* [54]. Marine trace fossil diversity increases through the Ediacaran-Cambrian boundary [5,55–60]. The vendobiont *Swartpuntia* persisted into Cambrian rocks of the Upper Member of the Wood Canyon Formation in the Mojave Desert, and the Poleta Formation in the Inyo Mountains [34]. Microbial mat textures also persisted into the Cambrian [61–62], and through to present day [63]. Early Cambrian archaeocyathids are found in local carbonate reefs within the Upper Member of the Wood Canyon Formation around Death Valley [64–66]. Early Cambrian marine fossils of the traditional *Olenellus* trilobite zone are found in the Carrara Formation [67–68], and correlative Latham Shale [68–73]. There are 4 successive biostratigraphic zones within these Cambrian formations [74–75], with meter levels above base of Carrara Formation in Emigrant Pass (Fig 3B) of 10 m for *Arcuolenellus arcuatus* zone, 18 m for *Bristolia mohavensis*, 34 m for *Bristolia insolens*, and 45 m for *Peachella iddingsi*. Donna Loy and Emigrant Pass Cambrian sections (Fig 3A-B) cap an Ediacaran succession, but the Cadiz section overlies the “Great Unconformity” [9–11] with underlying Mesoproterozoic (1.4 Ga) granite of the Mojave Province [76–77].

Carbon isotopic chemostratigraphy of the Wood Canyon Formation [78] has been used for an age model (Table 1) by correlation with isotopic minima dated radiometrically elsewhere [86,87]: 594 Ma for the carbon isotopic minimum immediately below the Stirling Quartzite contact (0 m in Fig 3A); 578 Ma for carbon isotopic minimum at 25 m in Stirling Quartzite (25 m), and 538.8 Ma for carbon isotopic minimum in the lower Wood Canyon Formation (106 m). Thus the section of Stirling Quartzite and Wood Canyon Formation near Donna Loy Mine (Fig 3A) has a geological duration from 594−536 Ma. Geological ages [87] for trilobite zones of the Carrara Formation are 513.1 Ma at 86 m (in Fig 3B) for *Arcuolenellus arcuatus*, 512.9 Ma at 94 m for *Bristolia mohavensis*, 512.6 Ma at 110 m for *Bristolia insolens*, and 512.4 at 121 m for *Peachella iddingsi*. An age model for Emigrant Pass section (Table 1) shows a geological age of the Zabriskie Quartzite and Carrara Formation of 514−512 Ma, so that these formations are disconformable on the Upper Member of the Wood Canyon Formation at Emigrant Pass (Fig 3B). Zabriskie Quartzite and Latham Shale near Cadiz (Fig 3C) have the same trilobite zones, so represent the same marine transgression as the Zabriskie Quartzite and Carrara Formation in Emigrant Pass.

**Table 1:** Equations for geological age and paleoenvironmental reconstructions of paleosols.

Equation	Input variables	R^2^	St.Dev.	P	Reference
*y *= −0.5117*x* + 592.61	Age (*y*, Ma) in Donna Loy section (Fig 3A) at height (*x*, m)	0.99	±1.65	3 ∙ 10^−6^	herein
*y *= −0.0197*x* + 514.78	Age (*y*, Ma) in Emigrant Pass section (Fig 3B) at height (*x*, m)	0.99	±0.019	4 ∙ 10^−9^	herein
$y=0.42e^{0.66x}$	Depth of burial (y, km) from Weaver index of illite crystallinity (*x*)	0.61	±0.57	0.01	[21]
$C=\frac{-0.51\times 100}{\left\{\left(\frac{0.49}{e^{\frac{B}{0.27}}}\right)-1\right\}}$	Compaction (*C*, fraction) due to depth of burial (*B*, km) by overburden	0	0	0	[79]
$\varepsilon_{i,w}=\left[\frac{\rho_{p}C_{j,p}}{\rho_{w}C_{j,w}}\right]-1$	Strain (ε) due to soil formation (mole fraction), immobile element (*i* = Ti, wt.%), bulk density (ρ, g.cm-3), oxide assay (*C*, wt, %), for elements (subscript *i,j*) of weathered material (subsript *w*) and parent material (subscript *p*).	0	0	0	[29]
$\tau_{j,w}=\left[\frac{\rho_{w}C_{j.w}}{\rho_{p}C_{j,p}}\right]\left[\varepsilon_{i,w}+1\right]-1$	Mass transfer (τ, mole fraction) due to soil formation, rest as above.	0	0	0	[29]
$\Delta_{WI}=B/K_{sample}-B/K_{Weaver}$	Adjusted B/K ratio ($\Delta_{WI}$), for B (ppm) and K (wt percent) analyzed for sample, and expected for a rock with the same Weaver illite crystallinity index	0.98	±2.9	0.0005	[30]
$A=3.92D^{0.34}$	Age (*A*, kyrs) of paleosol, diameter (*D*, cm) of pedogenic carbonate nodules	0.57	±1.8	0.001	[80]
A=3.987G+5.774	Age (*A*, kyrs) of paleosol, abundance of gypsum (*G*, % surface area).	0.95	±15	0.01	[29]
$P=137.24+6.45D-0.0132D^{2}$	Mean annual precipitation (*P*, mm), depth in profile to calcareous nodules (*D*, cm), corrected for burial compaction.	0.52	±147	0.0001	[80]
$P=87.593e^{0.0209D}$	Mean annual precipitation (P, mm), depth to gypsum (*D*, cm) corrected for burial compaction	0.63	±129	0.0001	[81]
*R* = 0.79*T* + 13.7	Seasonality or wettest minus driest month precipitation (*R*, mm), thickness of the calcic horizon (*T*, cm)	0.58	±22	0.0001	[80]
$CIA=\frac{mAl_{2}O_{3}\times 100}{mAl_{2}O_{3}+mCaO+mNa_{2}O+mK_{2}O}$	Chemical Index of alteration (*CIA*) from moles of major elements (*mAl*_*2*_*0*_*3*_*, mCaO, mNa*_*2*_*O, mK*_*2*_*O*) obtained by dividing weight percent by molecular weight	0	0	0	[82]
T=−18.5AI+17.3	Mean annual temperature (*T*, °C), ratio of soda+potash/alumina (*AI*, mole fraction alkali index)	0.37	±4.4	0.0001	[83]
T=0.21CIW−8.93	Mean annual temperature (*T*, °C), chemical index of weathering (*CIW* = CIA without K_2_O)	0.81	±2.1	0.001	[84]
$P_{r}=25.3D_{c}+588$	Soil respired CO_2_ (*P*_*r*_, ppmv), original depth to pedogenic carbonate corrected for burial compaction (*D*_*c*_, cm)	0.66	±768	0.0001	[85]
$P_{r}=42.9D_{g}+399$	Soil respired CO_2_ (*P*_*r*_, ppmv), original depth to pedogenic gypsum corrected for burial compaction (*D*_*g*_, cm)	0.64	±552	0.05	[85]

Sedimentological studies of the Stirling and Zabriskie Quartzites and parts of the Wood Canyon Formation have concluded that they were deposits of rivers, estuaries and beaches, with local tidal influence [3,12–17,88,89]. Cambrian trace fossils of *Skolithos* and *Arenicolites* in the Wood Canyon Formation have also been interpreted as estuarine, and an early animal invasion of fresh water [90], although not without dissent [16,17,91] Some late Cambrian and early Ordovician trilobites may have been estuarine as well as marine [92], and this possibility is examined here for trilobites in the Latham Shale and Carrara Formation. Archaeocyathids and cloudinids in the Upper Member of the Wood Canyon Formation are generally accepted as marine [65–66]. Quilted Ediacaran fossils such as *Ernietta*, *Swartpuntia*, and *Pteridinium* have traditionally been considered marine [23,24,33–37,44,50] although some vendobionts are now reinterpreted as non-marine [20–22,30,93–96]. Vendobiont habitats are one of the main questions tested here.

Plate tectonic reconstructions show that southeastern California was equatorial during the Ediacaran and Cambrian, on the northern margin of an east-west oriented Laurentian craton [97,98]. Paleopoles for the formations considered here [99,100] can be recalculated using standard methods [101,102] and yield tropical paleolatitudes of 3.6^o^ ± 7^o^ for the Bonanza King Formation, 4.4^o ^± 4^o^ for the Carrara Formation, 0.4^o^ ± 2^o^ for the Wood Canyon Formation, and 16.7^o ^± 7^o^ for the Johnnie Formation.

## Materials and methods

Ediacaran and Cambrian sections were examined at three localities in California: (1), near Donna Loy Mine starting at N35.812373^o^ W116.080104^o^ (Fig 3A), (2), in Emigrant Pass starting at N35.889276^o^ W116.076449^o^ (Fig 3B), and (3), 3 km northeast of Cadiz starting at N34.53564^o^ W115.47716^o^ (Fig 3C). Also studied were Ediacaran localities (4) in the Stirling Quartzite along the ridge 3 km southwest of Johnnie (N36.4047977^o^ W116.095326^o^), in Nye County Nevada. The Lower Member of the Wood Canyon Formation was examined (5) near a large cairn 4 km south of Johnnie (N36.391947^o^ W116.104116^o^), and at three fossil localities 3 km west of Johnnie, (6) at N36.4325647^o^ W116.1081905^o^, (7) at N36.1432118^o^ W116.1101740^o^ and (8) at N36.427285^o^ W116.111593^o^, all in Nye County, Nevada. Stratigraphic sections of individual beds were measured at centimeter scale, and oriented rock samples collected for laboratory studies, including bulk chemical composition (Supplementary Information S1 Table) and trace elements (S2 Table). Thin sections (Fig 5) were prepared as evidence for grain size (S3 Table) and mineral compositions (S4 Table) by point counting (500 points) with a Swift automated stage and Hacker counting box on a Leitz Orthoplan Pol research microscope. Accuracy of this many points (Figs 6–7) is ± 2% for common constituents [103]. Major and trace element chemical analysis was determined by XRF, and boron by ICP in fused beads at ALS Chemex in Vancouver, Canada. Bulk density was measured by the clod method, with three measurements of raw weight, and then clods coated in paraffin of known density in and out of chilled water [104]. Rare earth element (YREE) data were normalized to Post-Archean Australian shale (PAAS) values [105]. Boron data supplements a large dataset of comparable data [30] for determining paleosalinity of Ediacaran and Cambrian fossils and paleosols (S5 Table). Measurements of depth and thickness of calcic horizons were taken in the field as evidence for paleoclimate (S6 Table). Paleoclimate and phosphorus depletion of the paleosols also was inferred from chemical compositions within the profiles (S7 Table). Fossils and rock specimens used in this work are mostly conserved within the Museum of Natural and Cultural History of the University of Oregon, in Eugene, Oregon (online catalog paleo.uoregon.edu). Also figured are specimens from the Museum of Paleontology of the University of California at Berkeley, and the Department of Paleobiology of the Smithsonian Museum of Natural History. No permits were required for making these collections, which complied with all relevant regulations of the U.S. Bureau of Land Management.

**Fig 4:**
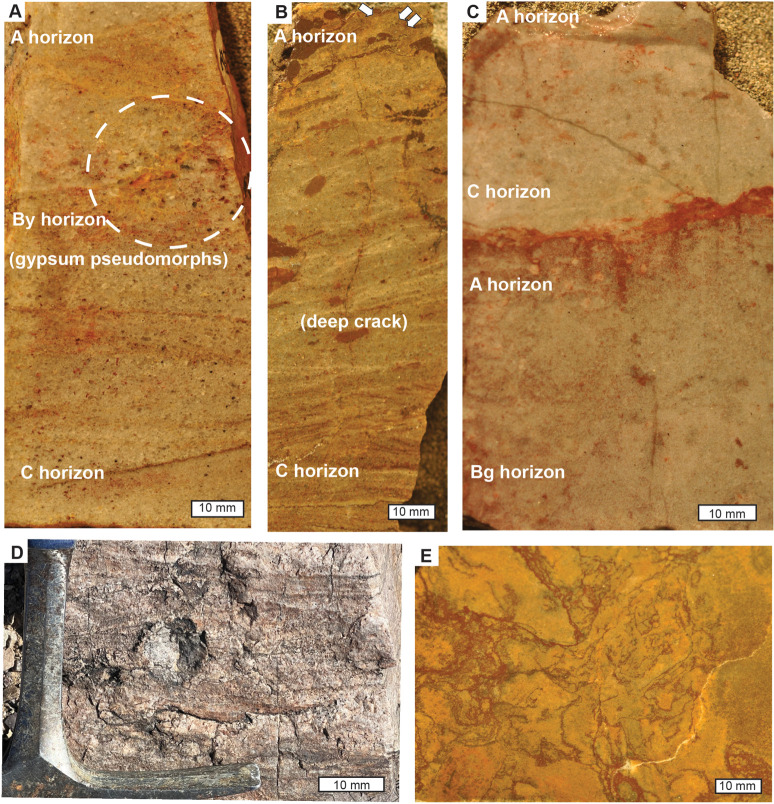
Polished slabs of paleosols and sedimentary beds: A, Hebinga loam paleosol in Stirling Quartzite Donna Loy Mine (45 m in Fig 3A), showing radial gypsum pseudomorphs; B, Duhubite loam paleosol with vesicular structure at arrows, in Wood Canyon Formation, Donna Loy Mine (79 m in Fig 3A); C, Buinga sandy loam paleosol, Zabriskie Quartzite, Cadiz (19 m in Fig 3C); D, radial gypsum pseudomorph in Hebinga paleosol 2 miles southwest of Johnnie; E, Nataanga loam paleosol in Wood Canyon Formation, Donna Loy Mine (99 m in Fig 3A), in vertical (D) and horizontal section (E).

### Metamorphic and diagenetic alteration

Paleosols in sedimentary rocks are ancient soils formed after deposition and before burial, so soil formation itself is a form of early diagenesis. Disentangling early and late diagenesis and metamorphism is needed for paleoenvironmental interpretation of paleosols. Rocks identified as paleosols in this study show two early diagenetic alterations: (1) drab mottles in upper parts of beds is often due to burial gleization of buried organic matter, and (2) dark red (Munsell 10R) color is commonly from dehydration reddening of ferric hydroxide minerals [106]. Burial gleization is chemical reduction of oxides and hydroxides of iron by anaerobic bacteria fueled by organic matter after subsidence into anoxic water: characteristic are drab mottles and tubular features radiating down from bed tops (Fig 2G), as in other Cambrian [107,108] and Proterozoic red beds [21,27,28,109]. Geologically ancient red beds are usually deep red in color from burial dehydration of ferric oxyhydroxides (Fig 2G), unlike brown to yellow modern soils and late Pleistocene sediments [106].

**Fig 5:**
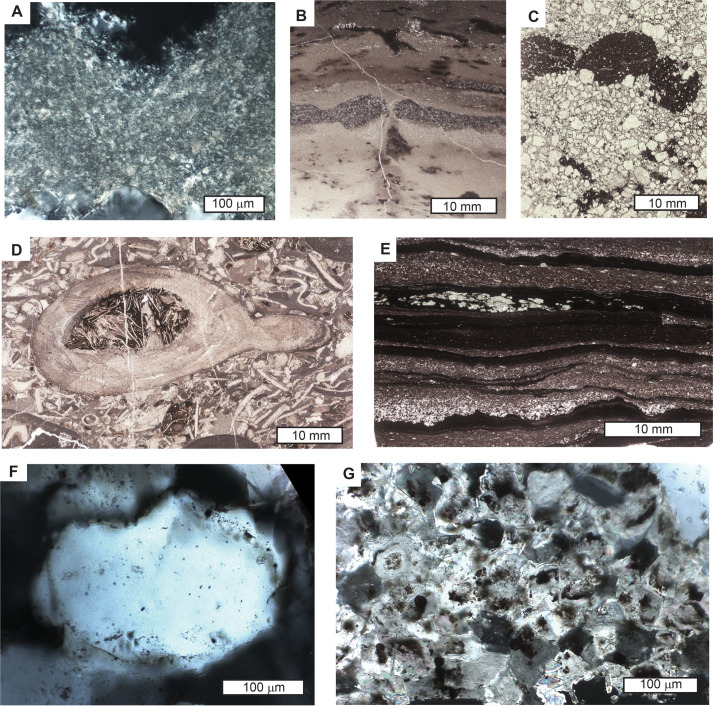
Oriented thin sections cut vertical to bedding: A, intertextic mosepic plasmic fabric in Bw horizon of Hebinga loam paleosol, Stirling Quartzite, near Donna Loy Mine (45 m in Fig 3A); B, surface cracking and oxidation, A horizon of Bui silty clay loam, Carrara Formation, in Emigrant Pass (72 m in Fig 3B); C, ferruginized dolostone clasts faceted on top, A horizon of Duhubite loam paleosol, Upper Member of Wood Canyon Formation, near Donna Loy Mine (79 m in Fig 3A); D, shell fragments in A horizon of Pakuitah silt loam paleosol, Chambless Limestone near Cadiz (42 m in Fig 3C); E, graded bedding within varves and soft sediment deformation preserved in C horizon of Wookki silty clay loam paleosol, Carrara Formation, in Emigrant Pass (71 m in Fig 3B); F, syntaxial quartz overgrowth cement on dusty rim of original grain, A horizon of Naatanga loam paleosol, Wood Canyon Formation, near Donna Loy Mine (99 m in Fig 3A);G, silt sized, subrounded rhombs of dolomite, A horizon of Duhubite loam paleosol. Wood Canyon Formation, near Donna Loy Mine (79 m in Fig 3A). Specimens in the Museum of Natural and Cultural History, University of Oregon, Eugene are A, R5354; B, R5832; C, R5852; D, R5816; E, R5844; F, R5356; G, R5852.

Section measuring adjusted vertical eyeheight differences by cosines [110] for dips of 31^o^E on strike azimuth 221^o^ for the sections at Emigrant Pass (Fig 2A), 50^o^E on strike 320^o^ near Donna Loy Mine (Fig 1B), and 12^o^E on strike 141^o^ for the base of the Cadiz section (Fig 3C). With highly variable K_2_O values of 0.1 to 7.76 wt % (S1 Table), there is no evidence of pervasive late diagenetic potash metasomatism, but some local illite deposition [111]. A Weaver index of illite crystallinity (ratio of 10/10.5Å peak in XRD trace) of 2.7–3.4 (S5 Table), is compatible with burial by 2.5–4.0 km [21]. Regional mapping reveals overburden above the Carrara Formation of at least 3 km, with perhaps an addition 1.5 km of eroded Permian to Jurassic, shown in a cross section for the southern Nopah Range including the Donna Loy Mine [112]. Burial compaction expected for 2.5–4.0 km burial is 57–63%, using a formula [79] in Table 1 with depth of burial, and physical constants appropriate to these materials. This degree of compaction is supported by ptygmatic deformation of clastic dikes in polished slabs (Fig 4B,E). Such compaction estimates are needed to calculate original depth to calcic or gypsic horizons in paleosols for paleoenvironmental interpretatiions (S6 Table). Compaction was accompanied by late diagenetic cementation with silica, including syntaxial authigenic overgrowths of quartz grains preserving original dusty rims (Fig 5F).

**Fig 6:**
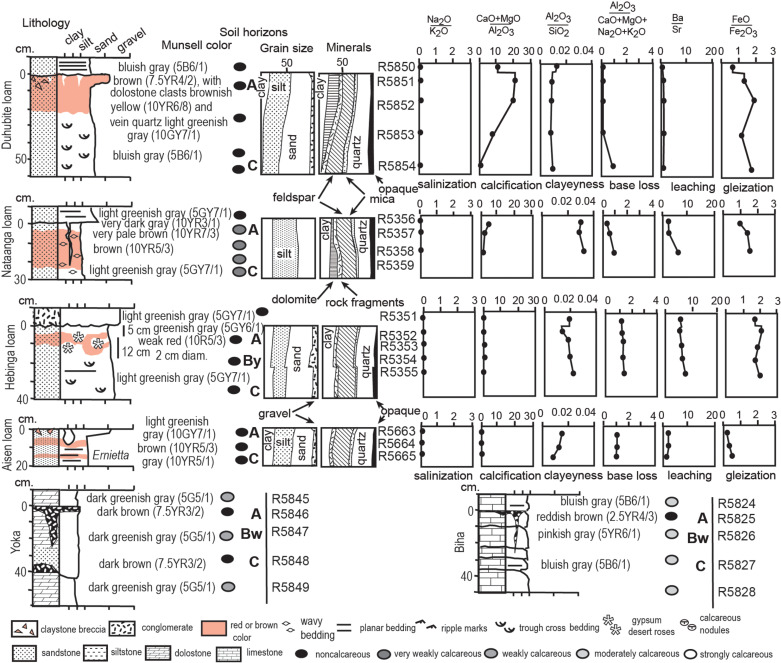
Ediacaran paleosol profiles their petrographic composition from point counting thin sections, and weathering trends revealed by molecular weathering ratios ( Tables S1-S2). Stratigraphic levels of these profiles are labelled in Fig 3. For field photos of two of the paleosols see Fig 2D-E, and for photomicrographs see Fig 5A, C, F-G.

**Fig 7:**
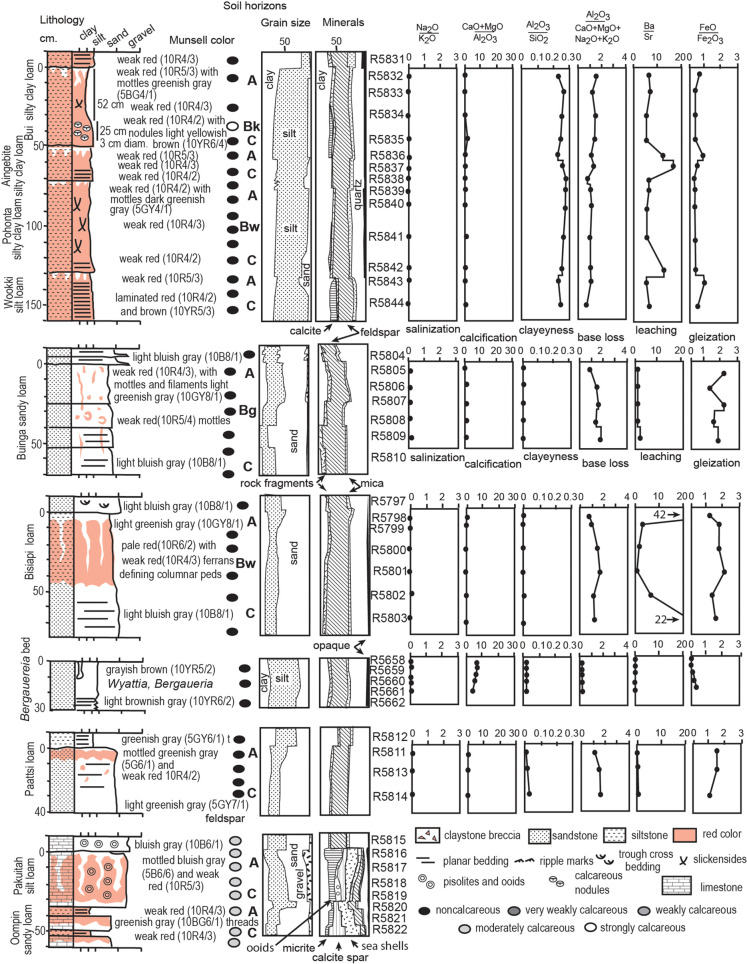
Cambrian paleosol profiles their petrographic composition from point counting thin sections, and weathering trends revealed by molecular weathering ratios ( Tables S1-S2). Stratigraphic levels of these profiles are labelled in Fig 3. For field photos of two of the paleosols see Fig 2F-G, and for photomicrographs see Fig 5B, D-E.

**Fig 8:**
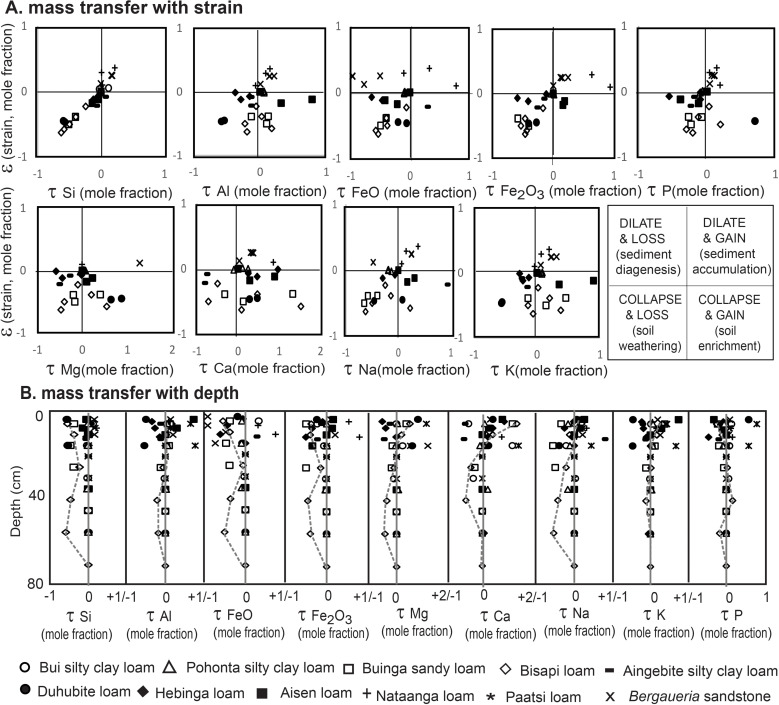
Tau analysis of Ediacaran and Cambrian paleosols of California, including elemental mass transfer versus strain **(A)**, and versus depth in paleosol profiles **(B)**.

**Fig 9:**
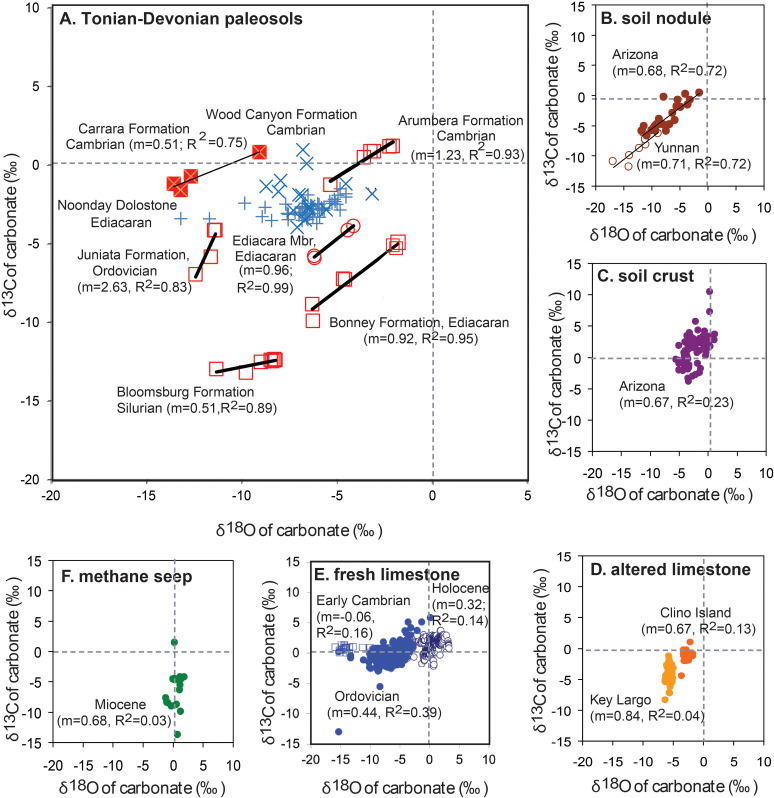
Covariance of carbon and oxygen isotopic composition of carbonate as a characteristic of paleosols, rather than other settings: A, paleosols of the Cambrian Carrara Formation [152], of the Ediacara Member of South Australia [140], of the Cambrian Arumbera Formation at Ross River [89], of the Ordovician Juniata Formation of Pennsylvania [26], and of the Silurian Bloomsburg Formation of Pennsylvania [25]; (B), soil nodules (above Woodhouse lava flow, near Flagstaff, Arizona [156] and in Yuanmou Basin, Yunnan, China [155]; (C), soil crusts on basalt (Sentinel Volcanic Field, Arizona, from [156]); (D), Quaternary marine limestone altered diagenetically by meteoric water (Key Largo, Florida, [139], and Clino Island, Bahamas, [158]); (E), Holocene (open circles) and Ordovician (open squares) unweathered marine limestones [154] and Early Cambrian (closed circles), Ajax Limestone, South Australia [153]; (F), marine methane cold seep carbonate, Miocene, Santa Cruz Formation, Santa Cruz, California [161] and Pliocene, Quinault Formation, Cape Elizabeth, Washington [162]. Slope of linear regression (m) and coefficients of determination (r^2^) show that carbon and oxygen isotopic composition is significantly correlated in soils and paleosols, but not in other settings.

**Fig 10:**
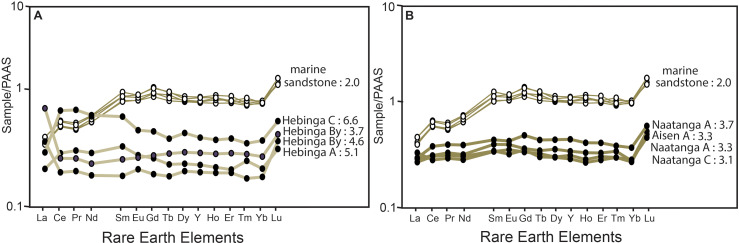
Rare earth element analyses of Ediacaran paleosols compared with Cambrian marine sandstone: A, Hebinga loam paleosol in Stirling Quartzite near Donna Loy Mine, and B, Aisen and Naatanga silt loam paleosols in the Lower Wood Canyon Formation near Donna Loy Mine, and multiple levels of sandstone bed with *Bergaueria* and *Wyattia* in Upper Member of the Wood Canyon Formation in Emigrant Canyon. This Aisen profile includes *Ernietta* in place (specimen F123791A). Numbers after the labels are LYREE/HYREE ratios, which are 3 or more for soils and paleosols, but less than 3 for marine rocks.

**Fig 11:**
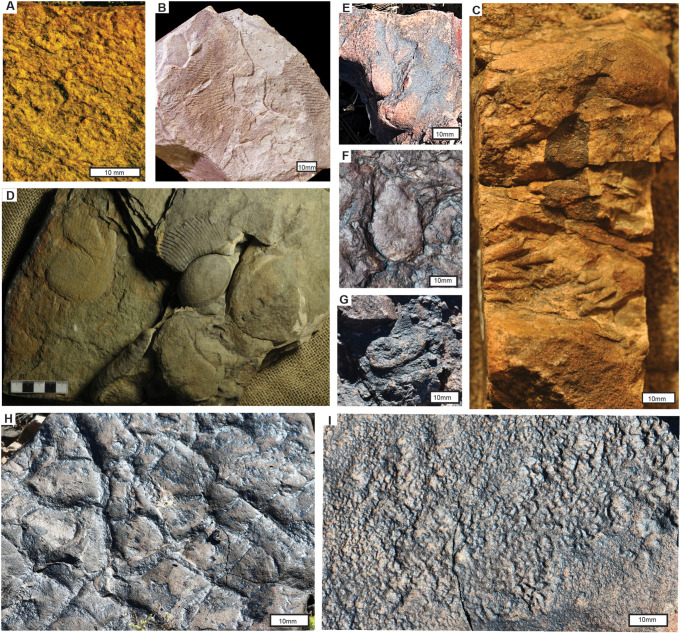
Ediacaran and Cambrian fossils and sedimentary structures of southern California: A, *Hallidaya brueri* discoids from Stirling Quartzite near Donna Loy Mine (45 m in Fig 3A); B, *Swartpuntia germsi* from Poleta Formation near Westgard Pass; C, *Bergaueria hemispherica* burrow and *Wyattia reedensis* conical hyoliths, from the Cambrian Upper Member of the Wood Canyon Formation in Emigrant Pass (21 m in Fig 3B); D, *Ernietta plateauensis* (sack shaped) and *Pteridinium simplex* (elongate and strongly fluted) showing pleating and basal seam at angle to bedding, from Lower Member of Wood Canyon Formation south of Johnnie; E-G, *Ernietta plateauensis* bulbs in life position (E), bulb and leaf bases on bedding plane (F) and leaves protruding for bed top among ventifacted pebbles (G) from Lower Member of Wood Canyon Formation west of Johnnie, Nevada; H, mudcurls within mudcracks (invalid trace fossil name “Manchuriophycus”) from west of Johnnie; I, *Rivularites repertus* microbial earth structure from west of Johnnie. Formations of these specimens are Ediacaran Stirling Quartzite **(A)**, Cambrian Poleta Formation **(B)**, Cambrian Upper Member of Wood Canyon Formation **(C)**, and Ediacaran Lower Member of Wood Canyon Formation (E-I). Specimen numbers are A, F123781 Museum of Natural and Cultural History, University of Oregon, Eugene; B, F37450 Museum of Paleontology, University of California Berkeley image courtesy of Dave Strauss; D, USNM 642300 Paleobiology Smithsonian Institution Museum of Natural History; C, E-G, Museum of Natural and Cultural History, University of Oregon, Eugene, F126060 **(C)**, F130202 **(E)**, F130207 **(F)**, F130201 **(G)**. H and I are field photos.

**Fig 12:**
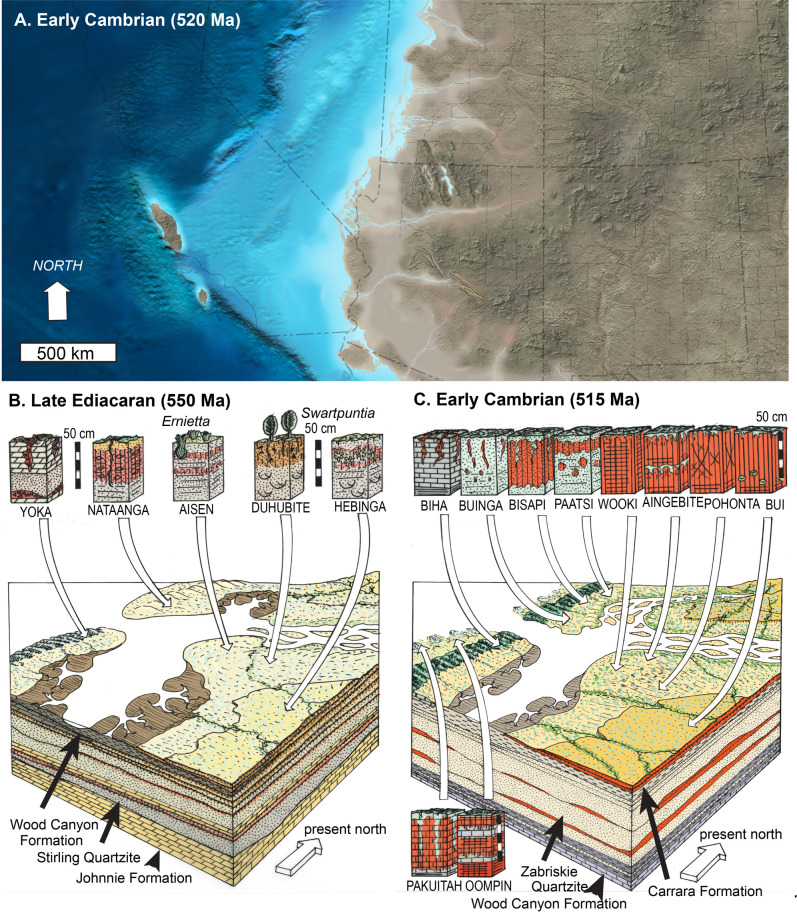
Paleogeographic map for 520 Ma (A) by Ron Blakey, and reconstructions of soils for Late Ediacaran (B, 550 Ma) deposition of Lower Wood Canyon Formation, and Cambrian deposition of Carrara Formation and Latham Shale (C, 515 Ma). Map reproduced from Colorado Geosystems Inc. under license #20110385-P. The map is in modern geographic coordinates with state outlines, but during the Ediacaran and Cambrian the coast was not north-south, but east-west near the equator (Torsvik and Cocks 2013; Scotese 2021).

## More abundant angular pebbles toward bed tops

### Observations

A surprising feature of some sandstone bed surfaces of the Lower Member of the Wood Canyon Formation is closely spaced and interlocking, matrix supported, angular pebbles of ferruginized dolostone and vein quartz toward the surface of some beds (Fig 2E). The clasts are well spaced within the bed but then become much more closely spaced to interlocking toward the top. Some clasts at the surface show planar facets (Fig 5C), and others are cracked but not displaced (Fig 4B). Both the pebbles and the sandstone are increasingly blackened by iron-manganese cement toward the top of the bed. This dark increasingly pebbly upper part of the bed contrasts with lighter colored sandstone below and above (Fig 2E). This upper part of trough cross bedded sandstones shows increased abundance of reddened dolostone clasts toward the top (Fig 6).

### Interpretation

Pebbly horizons within sandy fluvial strata are usually clast-supported, rounded pebbles or claystone breccia at the base of sandstone beds, because large clasts require greater energy for transportation in faster flow near the base of the bed [114,115], but these examples are at the top of the beds. Another possibility for these bed-topping clasts are stone lines, where an erosional plane is littered with pebbles, then covered again with finer grained sediment [116,117]. The examples here (Figs 2E, 4B, 5C) are not well spaced pebbles confined to a single plane, but show increasingly close spacing of clasts toward the top of the bed. A more likely explanation for these oversize clasts is desert pavement [118–120], known in Australia as gibber plain [121]. These form by eolian deflation of fine-grained matrix, so that stones too large to be lifted by wind settle on the surface [120]. Modern desert pavements are also formed by plant and animal dislocation of pebbles [120], but that would not have been a factor during Cambrian or Ediacaran periods. Wind scouring of surface pebbles creates planar facets of ventifacts (Fig 2E), and other pebbles are cracked thermally or physically (Fig 5C), without the embedded halves moving apart [122]. Rock varnish of iron-manganese (Fig 2E) and spherical cavities like vesicular structure (Fig 4B) are also features of modern desert pavements [118,119], but no calcrete or gypsum was seen in these Ediacaran examples. Vesicular structure of desert soils is formed after rainstorms when dormant microbes come to life and release oxygen and carbon dioxide bubbles into mud [118]. Interlocking ventifacted pebbles of gibber paleosols are also known in the Cambrian Flathead Sandstone of Montana [123], and the Triassic Budleigh Salterton Beds of England [122].

## Granulometry

### Observations

A surprise from point counting of what appeared to be red claystones of the Carrara Formation was that they had a very high proportion of as much as 79.8 volume % of angular silt grains of quartz and feldspar. These are not just parts of graded beds, but consistently silt-rich, massive beds (Fig 7), distinct from finely laminated interbedded units (Fig 5E). Thin section examination of brown siltstone of the Lower Member of the Wood Canyon Formation also reveals abundant, angular, silt size-grains of dolomite (Fig 6), along with granule and sand-size grains of quartz. The dolomitic silt is not interlocking crystals, but grains that are partly ferruginized and weathered, so evidently transported (Fig 5G). Also distinct from crystalline carbonates is the very high proportion of quartz and feldspar in these silty beds (Figs 6-7). These red and brown siltstone beds are distinct from sand to granule size of interlocking crystals of neomorphically recrystallized gray dolostones and limestones in the same section, and also contain mud cracks, and gypsum crystals as evidence of subaerial exposure (Fig 4A,D). The silt-rich beds also lack lamination or varves found in other stratigraphic levels (Fig 5B, E). The silt grains are angular and grain-supported rather than embedded in a clayey matrix (Fig 5G).

### Interpretation

A plausible explanation for these unusually silt-rich beds is that they were loess, deposited on land by wind. Much eolian silt may have fallen into the sea before landscapes stabilization by plants, but these examples are grain-supported (Fig 5G), not embedded in clay and carbonate, nor within graded beds, as found in Cambrian marine siltstones [124]. Silt-rich beds of the Lower Member of the Wood Canyon and the Carrara Formation are very similar in grain size, angularity, and texture to Quaternary Peoria Silt [125,126]. Eolian additions of dolomite are apparent in surface of some beds (Fig 6), comparable with Peoria Silt which has 42% carbonate at Vicksburg, Mississippi [127], 32% at Cumback, Indiana [128], and 31% in a core G56 in Illinois, 80 km north of St. Louis [129]. Such compositional differences in Peoria Loess reflect distance from freshly deglaciated Paleozoic limestones and dolostones. Other similar eolian loess deposits are the basal Ediacaran Nuccaleena Formation of South Australia [130], upper Moonlight Valley Tillite and lower Ranford Formation of Western Australia [93], Late Ediacaran Ediacara Member of South Australia [21,131], and Late Cambrian Mount Simon Sandstone of Illinois [132]. Some of these Ediacaran and Cambrian units have mistakenly been interpreted as marine [23,133,134].

## Crack patterns

### Observations

Cracks with v-shaped profiles have been illustrated previously in the Stirling Quartzite [15], and mud chips in the Zabriskie Quartzite [14]. Also present are near-vertical cracks filled with darker material from above, stained with iron and aluminum and folded by burial compaction below the laminated cover to massive beds (Figs. 4B-D, 5B). A horizontal view of one of these cracked bed surfaces shows complex deformation of the cracks filled with angular fallen pieces and minor slumps within a generally polygonal network (Fig 4E).

### Interpretation

The v-shaped cracks are like simple desiccation polygons [135], found in very weakly developed clayey soils exposed to air. With continued soil development, systems of desiccation cracks are modified by clay and oxidation on the surface (cutans) and these define characteristic units of soil structure (Fig 4D-E) known as blocky angular peds [95]. Marine or lacustrine shales, in contrast, have uncracked lamination or varves (Fig 5E). Some of the cracks are in sandy beds (Fig 4B,E), rather than clay usual for desiccation cracks [135]. The enigma of sand cracking like clay has been explained [136] as due to abundant hydrated microbiota, like that of a microbial earth soil [137]. An alternative explanation may be frost cracking in a periglacial soil [138,139], however, ice wedges are much larger and more strongly tapering than these examples (Fig 4B).

## Sand crystals

### Observations

Polished slabs and thin sections of the upper Stirling Quartzite revealed 1–2 cm diameter rosettes of sand crystals, radiating poikilotopic crystals (Fig 4A,D), at two stratigraphic horizons (Fig 3A). The crystals are less ferruginized than the matrix, are narrow and monoclinic, but consist of silica cement like the rest of the slab. They do not have the crystal form of chalcedony or quartz, and are likely pseudomorphs of a salt, replaced by the same deep burial silicification that produced quartz overgrowths (Fig 5F).

### Interpretation

These rosettes are identical to gypsum sand crystals commonly known as desert roses [140,141], which form by replacement and cementation without extensive displacement of matrix within the confining pressure of desert soils and playa lake beds. Such replacive crystals with enclosed matrix grains are very distinct from limpid crystals and seams of gypsum and anhydrite in marginal marine or lacustrine sabkhas, where higher water content allows clear crystals precipitating from solution to displace surrounding saturated sediment [142,143]. As in gypsic (By) horizons of desert soils [81], desert roses of the Stirling Quartzite are organized into horizons a set distance below the top of the bed (Fig 4A,D).

## Calcite nodules

### Observations

Testing of freshly broken surfaces in the field with dilute HCl showed strong effervescence on nodular horizons in the lower Carrara Formation (Fig 7). These nodules of micritic, low-magnesium calcite are up to 3 cm in diameter and range in shape from spherical to irregularly ellipsoidal and lumpy. In thin section, their micritic cement is replacive, leaving relict grains mainly of strongly etched quartz. The nodules stand out as patches of yellow, hard, erosion-resistant mounds in the red clayey siltstone (Fig 2G), within a well-defined band a consistent distance below the erosional top of each massive bed (S6 Table).

### Interpretation

Micritic nodules aggregated into subsurface horizons (Fig 2G) with replacive, micritic microtexture and circumgranular cracks are most like calcic (Bk) horizons of soils [80]. Marine or lacustrine carbonates of the Wood Canyon and Carrara Formation with trace fossils and trilobites are very different: dolomitic, grey in color, laminated, and form beds with paleokarst [144,145] of red dissolution features on the upper surface (Fig 3,6).

## Mineral and chemical trends within beds

### Observations

Clay is enriched toward the top of most beds, and point counting reveals that enrichment is at the expense of feldspar and rock fragments (Figs 6-7). This petrographic trend is marked for beds with low molar ratios of ferrous over ferric iron, but less marked for beds with molar ratios of ferrous over ferric iron greater than 1. None of the beds are enriched in soda or barium near the top compared with the base, and only two show alkali and alkaline earth enrichment toward the top from dolomitic silt interpreted here as eolian additions (Figs 6-7). Unlike the other beds sampled, the *Bergaueria* bed of Emigrant Pass (Fig 7) shows little change from top to bottom.

### Interpretation

Alkali earth, alkali and strontium depletion along with surface clay enrichment at the tops of most beds are evidence of weathering by carbonic acid hydrolysis. The effect is most marked in beds with low ferrous/ferric ratios indicative of free drainage, and not so strong in beds with ferrous/ferric ratio greater than 1, due to waterlogging and chemical reduction. Clayey bed tops were not part of graded beds such as turbidites deposited in a water column [146,147] for the following reasons: clay is nowhere dominant (Figs. 6-7), colors are red (Fig 4), loess-like silt-dominated grain-size throughout bed (Figs. 6-7), and lack of surface-enrichment in alumina, lime and magnesia (Figs. 6-7). Asymmetric enrichment of clay in bed tops over intervals of only 15 cm is very different from symmetrical hydrothermal or diffuse metamorphic alteration [148,149]. Like other Cambrian and Ediacaran paleosols [21,93,96,108] these California and Nevada paleosols lack differentiation of subsurface clayey (Bt) horizons, which coevolved with Devonian trees [150,151].

The *Bergaueria* bed contains fossils known to be marine fossils, *Bergaueria hemispherica* [55] and *Wyattia reedensis* [46,152], unlike the other beds sampled (Figs. 6-7). This may explain lack of geochemical and textural differentiation in the *Bergaueri*a bed, unlike the other beds.

## Within bed geochemical mass balance

### Observations

A definitive method to disentangle soil formation from sedimentation is geochemical mass balance, or tau analysis, a reformulation of classical economic geology equations of ore mineral alteration [29]. Tau analysis distinguishes strain and transport aspects of alteration: (1) losses of mobile elements as mole fraction mass transport (τ_j,w_) and (2) bed swelling or shrinking as mole fraction strain (ε_i,w_) from an immobile element (Ti used here), using the formulae in Table 1. Soils and paleosols lose mass with weathering, along with nutrient cations and silica, so they have negative strain (ε_i,w _< 0), and have negative mass transfer (τ_j,w _< 0). In contrast, sediment accumulation, diagenetic alteration, and ore mineralization have positive strain and mass transfer because they add both elements and mass. Negative strain is partly offset by burial compaction [153,154]. Tau analysis of beds covers only the few decimeters between the sediment at the base of the bed as a parent material to the weathered top of the bed, and not large scale alteration of thick sequences of sedimentary rocks. Nor does tau analysis include weathering in hinterlands producing that sediment.

### Interpretation

Analyzed beds of the Stirling and Zabriskie Quartzites and Wood Canyon and Carrara Formations show mainly pedogenic alterations, with most data within the collapse and loss quadrant (Fig 8A). Beds in the sedimentary gain quadrants include the *Bergaueria* bed as well as Paattsi and Naatanga beds with relict bedding, and other indications of weak pedogenic differentiation. Limited chemical weathering and depletion of mass and weatherable elements (Fig 8B), is compatible with limited petrographic differentiation within the same beds (Figs. 6-7) and relatively weak soil development.

Tau analysis has been widely used for Precambrian paleosols [21,93,153], as well as Cenozoic paleosols [153,155], and modern soils [29,156], because it discriminates effectively between soil formation and sedimentation.

## Stable isotopic covariance

### Observations

Micritic low-magnesium calcite nodules of the Carrara Formation have stable isotopic composition [113] with strong covariance of δ^13^C and δ^18^O (Fig 9A). However, dolomitic silt of the Wood Canyon Formation (x in Fig 9A) and Ediacaran Noonday Dolostone (+ in Fig 9A) in the same area [113] shows no such correlation.

### Interpretation

Cross-plots of δ^13^C and δ^18^O in marine carbonate are widely used to screen for diagenetic alteration, which includes soil formation and karst weathering [28]. Unaltered marine limestone and sea-shells (Fig 9E) does not show any correlation [157,158]. The most profoundly altered carbonate is pedogenic, precipitated during early diagenesis between episodes of deposition and burial of alluvial sediment [106,80]. Significant (P > 0.05) covariance of δ^13^C and δ^18^O of carbonate is pronounced in Holocene soils (Fig 9B) in China [159] and Arizona [160], and in other Cambrian and Neoproterozoic paleosols [96,145,113,161]. Less significant correlations (Fig 9C-D) are found in soil carbonate crusts [160], and in marine limestone altered by deep circulation of meteoric water [144,162]. Only seasonally dry lake carbonates show covariant stable isotopes, from soil formation on the dry lake bed, but covariance is not seen in perennial open system lakes [163]. Methanogenic microbes create near constant δ^18^O but highly varied δ^13^C (Fig 9F) in carbonate of marine methane seeps [164–166], and in siderite of wetland paleosols [167,168]. From this perspective, low-magnesium calcite nodules in Carrara Formation have isotopic covariance like pedogenic nodules, but dolomite in the Wood Canyon and Noonday formations is like marine dolostones.

Strong correlation of δ^13^C and δ^18^O is caused by selection for light isotopologues of gaseous CO_2_ during photosynthesis passed on to soil respiration [31,169], whereas oxygen of water is vastly in excess of carbon in the sea or lakes [28]. Kinetic evaporative effects in narrow spaces of soils may promote correlation [170], but the major fractionation is due to stomatal conductance and then enzymes such as rubisco [28] and carbonic anhydrase [171]. The δ^13^C and δ^18^O covariance is also seen in both respired soil CO_2_ [172,173], and in plant cellulose [174,175]. Cambrian and Ediacaran paleosols predate the evolution of stomates, so enzymatic control is more likely responsible for observed covariance (Fig 9A). Such covariance is not affected by metamorphic alteration as high as greenschist facies in paleosols of the Juniata and Bloomsburg Formations (Fig 9A) [25–26], so is unlikely to have been altered by late diagenesis of the Carrara Formation.

## Rare earth element (YREE) composition

### Observations

Four beds were analyzed for yttrium and rare earth elements (YREE) including the *Bergaueria* bed which is very different from the others. The *Bergaueria* bed has LYREE/HYREE weight ratio of 2.0 throughout, but the others range from 3.1 to 6.6 (Fig 10). The *Bergaueria* bed is heavy-YREE-enriched and shows no internal differentiation compared with the other beds, which are light-YREE-enriched and have strong internal differentiation within the Hebinga bed, less marked in Aisen and Nataanga beds. The Hebinga bed is in the Stirling Quartzite, but very similar to Aisen and Nataanga beds in the Wood Canyon Formation (Fig 3A-B), so had a source area indistinguishable by YREE composition [14–15], though distinguishable in detrital zircon age spectra [176,177].

### Interpretation

Soils and granites are enriched in light YREE, with atomic numbers 57–62, rather than heavy YREE, with atomic numbers 63–71 so that LYREE/HYREE weight ratios (L/H of Table 1) are 3–24 [178,179]. In contrast, marine clays have YREE arrays with positive slope, and LYREE/HYREE ratios less than 3 [31,180]. Hydrothermal alteration like that of black smokers on the deep-sea floor creates anomalous enrichments of europium [181]. Similar YREE arrays have persisted in Archean (3 Ga) rocks despite metamorphism high in the greenschist facies and near total cementation and replacement by silica, which reduced overall YREE concentrations [182]. These values in ppm were normalized to Post Archean Australian Shale (PAAS) values [104] for plotting (Fig 10). A variety of ways of characterizing the slope of normalized YREE arrays were attempted, but the best discriminator from multiple trials was the simple ratio of non-normalized weights (ppm) of light YREE to heavy YREE (L/H of Table 1). Yttrium (Y) in the array was placed between Dy and Ho based on its effective ionic radius [183]. Calibration of Ediacaran to Cambrian arrays for this new weight ratio proxy came from a literature compilation of 471 YREE analyses from a variety of Holocene soils and sediments [31].

The Hebinga bed shows overall YREE depletion from the bottom to the top of the bed, a distance of only 30 cm, which is the opposite of soils with YREE-enrichment compared with parent materials [178,184–186]. Similar depletions during soil formation are seen in deep tropical soils [187,188], but these are many meters thick, and more deeply weathered profiles than the Hebinga bed. Comparable differentiation over only a few centimeters is found in acid-sulfate soils [189], and in acid-sulfate leaching around hydrothermal pools of volcanoes [190]. There is no evidence of hydrothermal or volcanic alteration of the Hebinga bed, because it lacks hydrothermal europium anomalies [182]. Acid-sulfate weathering of the Hebinga bed is indicated by desert roses (Fig 4A,D) like those of modern gypsic soils [140,141], and thus formed over many thousands of years [21]. In contrast Aisen and Naatanga beds show less internal differentiation (Fig 10B), because of a short time of weathering revealed by persistence of bedding (Fig 4E). All the putative paleosols have YREE patterns unlike the *Bergaueria* marine bed, which shows no internal differentiation and marked heavy-YREE enrichment (Fig 10). Heavy YREE enrichment has been observed in transition from modern fluvial to marine sediments [191,192], and also used to discriminate Precambrian freshwater from marine rocks [193].

## Boron content

### Observations

Boron content of sedimentary rocks can be used as a paleosalinity proxy because marine waters have high boron content of 20–50 ppm, whereas freshwater has only 2 ppm [194]. Diagenetic illitization depletes boron in deeply buried Paleozoic and Precambrian rocks [195], and metamorphism further depletes boron [196]. The critical value of boron/potassium (B/K) dividing marine from non-marine declines predictably with increased Weaver index of illite crystallinity [30]. An adjusted B/K ratio ($\Delta_{WI}$ ) can be calculated by the formula in Table 1, with values that are low (±2.9) for estuarine rocks and fossils. The adjusted B/K ratio ($\Delta_{WI}$) is negative for non-marine and positive for marine (S6 Table).

### Interpretation

Californian specimens (S6 Table) of a stromatolite (*Boxonia*), alga (*Elainabella*), worm tube (*Conotubus*) and discoid (*Beltanelliformis*) had positive adjusted B/K ratio, so turned out to be marine as was expected from their bedded shale or limestone matrix. Also marine was a trilobite (*Olenellus*) from Cadiz, which had such a low positive value that it may have been estuarine. This observation supports the idea of early animal invasion of fresh water [90], which has been controversial [91]. Late Cambrian and early Ordovician trilobites were also estuarine, but there is little doubt that the exposed gills of trilobites precluded extended excursions onto land [92]. All the other fossils in S6 Table had non-marine B/K, and were in putative paleosols (Fig 3. Similar fossils from Ediacaran rocks of Namibia (S6 Table) also had nonmarine B/K [30].

## Paleosols

### Paleosol recognition

Many of the features discussed in preceding paragraphs are evidently non-marine and pedogenic. The most diagnostic field criterion for paleosols is fossil root traces [106], but Ediacaran and Cambrian rocks are much older than vascular land plants [25–26], so other features must be used. Especially useful are soil cracking structures and pseudomorphs of soluble salts, seen in polished slabs (Fig 4A) and outcrops (Fig 4D). Disconformities and massive beds without obvious sedimentary structures are candidates for paleosols in Ediacaran and Cambrian fluvial facies, and are widely recognized in southern California and Nevada [3,12–17,60,88]. The examined sections also include marine rocks and fossils [46,48,49,75]. Sedimentological criteria alone are usually inadequate to distinguish Cambrian marine and non-marine rocks [197].

### Paleosol classification

The preceding paragraphs described a variety of paleosol features in Ediacaran and Cambrian rocks of southern California, but the rest of this paper explores the kinds of paleosols present and their paleoenvironmental implications (Fig 12). Many beds analyzed as putative paleosols have been given non-genetic names (Table 2) using the Shoshoni native American language [203]. These pedotypes can be interpreted in terms of soil taxonomy to build a model of their paleoenvironments (Table 3). Hebinga profiles with cracked surface (A horizon) over a diffuse horizon with mottles and sand crystals (By or gypsic) are most like Gypsids [198]. Bui profiles, on the other hand, have green mottled surfaces (A horizon) over an horizon of pedogenic carbonate nodules (Bk or calcic), as in Calcids [198]. Pohonta profiles have a cracked and mottled surface (A) over slickensided clay (Bw) as in Vertisols [198]. Other profiles are less well developed, most like Entisols and Inceptisols [198], and would have been restricted to disturbed parts of the landscape (Table 3). The same criteria can be used to classify these paleosols in other classifications (Table 2) of Australia [201,202], and of the Food and Agriculture Organization [199,200].

**Table 2:** Pedotypes and diagnosis for Ediacaran-Cambrian paleosols from California.

Pedotype	Meaning	Diagnosis	USDA [198]	FAO [199,200]	CLASSIC [201]	AUSTRA-LIAN [202]	Locality
Aisen	grey	Grey sandstone (A) over grey bedded sandstone (C)	Aquent	Dystric Fluvisol (Jd)	Alluvial soil	Stratic Rudosol	Donna Loy
Aingebite	Red	Thin, red-green mottled claystone (A) over bedded shale (C)	Ochrept	Dystric Cambisol (Bd)	Brown clay	Dystric Tenosol	Emigrant Pass
Biha	crack	Red stained cracks and rubble (A) into grey dolostone (C)	Orthent	Lithosol (I)	Terra rossa	Lithocalcic Calcarosol	Emigrant Pass
Bisapi	face paint	Red-green mottled sandstone (A) over red sandy (Bw) and grey sandstone (C)	Ochrept	Dystric Cambisol (Bd)	Red clay	Dystric Tenosol	Cadiz
Bui	eye	Grey-red mottled siltstone (A) over subsurface micritic nodules (Bk) on red shale	Calcid	Calcic Xerosol (Xk)	Red calcar-eous soil	Calcic Calcarosol	Emigrant Pass
Buinga	green	Greenish-gray sandstone with ferruginous tubules (A) over greenish grey sandstone with red mottles (Bw) and grey sandstone (C)	Aquept	Dystric Gleysol (Gd)	Humic Gley	Organosol	Cadiz
Duhubite	black	Purple-black disrupted sandstone (A) over cross-bedded sandstone (C)	Psamment	Eutric Regosol (Re)	Earthy sand	Chemic Tenosol	Donna Loy
Hebinga	flower	Grey sandstone (A) over red mottled sandstone with spherulitic gypsum pseudomorphs (By) and bedded sandstone (C)	Gypsid	Orthic Solonchak (Zo)	Solonchak		Donna Loy
Nataanga	orange	Orange disrupted dolomitic sandstone (A) over grey bedded sandstone (C)	Fluvent	Eutric Fluvisol (Je)	Alluvial soil	Stratic Rudosol	Donna Loy
Oompin	Sand grains	Red-green mottled limestone (A) over oolitic limestone (C)	Fluvent	Eutric Fluvisol (Je)	Alluvial soil	Stratic Rudosol	Cadiz
Paattsi	shallow	Red-green mottled sandstone (A) over bedded gray sandstone (C)	Psamment	Dystric Fluvisol (Jd)	Siliceous sand	Arenic Rudosol	Cadiz
Pakuitah	cracked	Rubbly red limestone (A) over mottled red-gray limestone (Bw) and gray limestone	Orthent	Lithosol (I)	Terra Rossa	Lithocalcic Calcarosol	Cadiz
Pohonta	thick	Green-red mottled surface (A) overthick red slicksided claystone (Bw) and red shale (C)	Chromudert	Chromic Vertisol (Vc)	Red Clay	Red Vertosol	Emigrant Pass
Wookki	striped	Green mottled siltstone (A) over red bedded siltstone (C)	Fluvent	Dystric Fluvisol (Jd)	Alluvial soil	Stratic Rudosol	Emigrant Pass
Yoka	Cave in	Brown stained cracks and rubble (A) into grey dolostone (C)	Orthent	Lithosol (I)	Terra rossa	Lithocalcic Calcarosol	Emigrant Pass

**Table 3:** Interpretation of pedotypes for Ediacaran-Cambrian paleosols from California.

Pedotype	Paleoclimate	Ecosystems	Parent Material	Palaeotopography	Years for formation
Aisen	Not diagnostic for climate	Microbial earth with *Ernietta* and *Pteridinium*	Quartz sand	Poorly drained near-stream levee	10-100
Aingebite	temperate (mean annual temperature 10.2 ± 2.1^o^C).	Microbial earth	Quartztofeldspathic silt	Well drained near-stream levee	100−1,000
Biha	Subhumid temperate	Microbial earth	Grey limestone	Beachrock ridge	1,000-100,000
Bisapi	temperate (mean annual temperature 8.9 ± 2.1^o^C).	Microbial earth	Quartzofeldspathic sandstone	Well drained levee	2,000-3,000
Bui	Semiarid (mean annual precipitation, 403–636 ± 147 mm), mean annual range of precipitation (27–69 ± 22 mm), temperate (mean annual temperature 9.7 ± 2.1^o^C).	Microbial earth	Quartzofeldspathic sandstone	Streamside swale	5,507−16,522 ± 1.8
Buinga	temperate (mean annual temperature 7.2 ± 2.1^o^C).	Microbial earth	Quartsofeldspathic silt	Coastal lagoon margin	2,000-3,000
Duhubite	Not diagnostic for climate	*Ernietta*, *Swartpuntia*, *Pteridinium* polsterland	Quartzofeldspathic sand and gravel	Estuarine levee	1,000-2,000
Hebinga	Arid (mean annual precipitation 267–288 ± 129 mm), temperate (mean annual temperature 10.1 ± 2.1^o^C).	*Hallidaya* posterland	Quartzofeldspathic sand	River levee	9,124−10,062 ± 15
Oompin	Not diagnostic for climate	Microbial earth	Oolitic limestone	Calcareous and bar	10-100
Paattsi	Not diagnostic for climate	Microbial earth	Quartzofeldspathic sandstone	Well drained levee	10-100
Pakuitah	Subhumid temperate	Microbial earth	oncolitic limestone	Beach ridge	2,000-3,000
Pohonta	Subhumid seasonal temperate (mean annual temperature 9.2 ± 2.1^o^C).	Microbial earth	Quartzofeldspathic silt	Well drained floodplain	6,000-10,000
Wookki	Not diagnostic for climate	Microbial earth	Quartzofeldspathic silt	Well drained near-stream levee	10-100
Yoka	Subhumid temperate	Microbial earth	Grey dolostone	Beachrock ridge	140,000-4,200,000

The best developed paleosols in each association of pedotypes represent stable landscape soils. In the FAO map classification [199,200] the Hebinga pedotype of the Ediacaran Stirling Quartzite was Orthic Solonchak, and would represent a map code of Zo + Re (Table 2). There is no comparable map unit of soils in North America [200], but similar is map unit Zo1-2a + Yh of the montane desert of coastal northern Chile and southern Peru between Punta Los Lobos and Antofagasta [199]. At Antofagasta, Chile, mean annual temperature is 16.9^o^C and mean annual precipitation is 148 mm [204]. The Duhubite pedotype of the Ediacaran Wood Canyon Formation was a Eutric Regosol, in a map code Re + Je, Jd, most like map unit Re5-1a + J,Yh on the coastal plain of northern Peru and southern Bolivia [199]. At Sechura, Peru, mean annual temperature is 23.6^o^C and mean annual precipitation is 30 mm [204]. The Bisapi pedotype of the Cambrian Zabriskie Quartzite was Dystric Cambisol, and would represent a map code of Bd + Gd, Jd (Table 2), most like Bd6-3a + Nd, I on the coast of Chile [199]. At nearby Concepción, Chile, mean annual temperature is 13.3^o^C and mean annual precipitation is 839 mm [204]. The Bui pedotype of the Cambrian Carrara Formation was Calcic Xerosol, and would represent a map code of Xk + Vc, Bd, Jd (Table 2), most like Xk1-2a + I on the coast of Chile [199]. At nearby Coquimbo, Chile, mean annual temperature is 16.3^o^C and mean annual precipitation is 104 mm [204]. These modern comparisons provide a general idea of Ediacaran and Cambrian paleoenvironments, and the following paragraphs evaluate each of these interpretations in detail.

### Original parent material

Parent materials to Ediacaran and Cambrian paleosols of southern California were mainly quartzofeldspathic sand (Figs 5C, 6,7) of granitic provenance [12–15,88,205]. Paleokarst paleosols formed by dissolution and ferruginization of marine limestone and dolostone, were not studied petrographically (Fig 6), with the exception of Oompin and Pakuitah profiles, which were partly marine because crowded with shell fragments and oolites (Fig 5D, 7). Some Ediacaran paleosols (Duhubite, Nataanga) also had substantial amounts of dolomitic silt with the grain size, angularity and bedding character of loess (Fig 5G), like other Ediacaran eolian deposits [21,45,130,131]. Clay may also have been included in this eolian parent material, because there is little evidence for clay production in most profiles from abundance of weatherable minerals or alumni/silica and alumina/bases ratios (Figs 6-7), as in soils near the arid-hyperarid transition in Chile [206]. Loess plains are among the most productive of modern soil parent materials, rich in weatherable minerals and physically stable [125,207].

### Reconstructed sedimentary setting

Fluvial paleocurrents from cross-bedding in the Stirling and Zabriskie Quartzites, Wood Canyon and Carrara Formations are from the southeast and south [13–17,88]. These streams drained nearby uplands of Mesoproterozoic (1.4–1.7 Ma) granite and gneiss of the Mojave Province [76–77]. These source areas were neither steep, nor cliffed, because fluvial conglomerate is rare, with only small pebbles, and in thin beds. The coastal plain had low gradient, because it was inundated by shallow sea several times in Lower and Upper Members of the Wood Canyon Formation, Carrara Formation and Latham Shale [2,67–70,73]. Another indication of low topographic gradient is the high FeO/Fe_2_O_3_ ratio of many, but not all parts of the paleosols (Figs 6−7), an indication of saturation by water table and dysaerobic conditions in the paleosols at depths of only 20−120 cm (after burial compaction) from the surface. Carbonate nodules (Bui pedotype), desert roses (Hebinga), and deep slickensides and clastic dikes (Pohonta) are evidence of well drained parts of paleosols on stable floodplains and terraces. Also found in well drained paleosols was loess inferred from grain size distributions, mineral contents (Figs 6−7), and angularity of siltstones (Fig 5G), comparable with modern loess [125,126,208]. Loess today forms distinctive landscapes characterized as “rolling downs”, but with steep angle of repose of terraces and erosional gullies near streams, because of self-support by angular silt grains [209]. Other paleosols with weak development and relict bedding (Aisen, Nataanga, Wookki, Aingebite), are in heterolithic sedimentary facies characteristic of streamsides and levees frequently disturbed by flooding [16–17]. Deep ocean was toward the northeast at Johnnie, Mt Dunfee and Westgard Pass (Fig 1), where there are thick marine shales and fossiliferous limestones [35,48,65,66].

### Time for formation

The cumulative time over which individual paleosols form can provide information on sediment accumulation rates of successions of paleosols. Duration of soil formation for Hebinga and Bui pedotypes can be calculated from chronofunctions for modern aridland soils. Diameter of pedogenic-carbonate nodules is related to radiocarbon age of nodules near Las Cruces, New Mexico [80], using an equation in Table 1. Similarly, abundance of gypsum in a profile, % surface area using a comparison chart [210], is a metric for soil age in the Negev Desert of Israel [21], according to equation in Table 1.

The New Mexico calcic chronofunction applied to eight Bui paleosols gave durations of 5.5 ± 1.8 kyr ranging up to 16.5 ± 1.8 kyr (S6 Table). Means and standard deviation for durations of all eight paleosols are 12.4 ± 3.9 kyr. The calcic Bui profiles show increasing duration up-section within three subunits of the Carrara Formation, each culminating in a marine limestone above red siltstones. Thus, lower sediment accumulation rate culminated in marine incursions. The Negev gypsic chronofunction applied to two Ediacaran Hebinga paleosols gave durations of 9.1 ± 15 kyr ranging up to 10.0 ± 15 kyr (S6 Table). This indicates a similar slow rate of subsidence in the southern Nopah Range localities of Emigrant Pass and Donna Loy mine from Ediacaran to Cambrian.

Other paleosols lack nodules or sand crystals, and retain original bedding below depths of 50 cm (Pohonta), 30 cm (Bisapi, Buinga, Pakuitah), 20 cm (Duhubite), 10 cm (Aingebite), or 1 cm (Aisen, Nataanga, Oompin, Paattsi, Wookki). The last category corresponds to 10–100 years of soil formation and the first category to 6,000–10,000 years (S6 Table). These are maximal estimates, because based on comparison with homogenization of bedding in Pleistocene soils of the San Joaquin Valley, California [211], which were more actively rooted and burrowed than Ediacaran or Cambrian soils. Agreement of the degree of pedogenic reworking in thick paleosols (Pohonta) and calcareous nodule paleosols (Bui) for which estimates of duration are available (S6 Table), suggest that rate of homogenization of these paleosols is not greatly different from modern, and thus impressive for the Ediacaran and Cambrian.

Paleokarst paleosols on limestone and dolostone (Biha and Yoka) also represent breaks in sedimentation. Maximum potential rates of limestone dissolution range from 5–404 mm/kyr, but are only 10–30 mm/kyr in semiarid regions [212] where there are Solonchaks and Calcids comparable with Hebinga and Bui paleosols. Dolostone dissolves at rates 3–60 times slower [213]. Thus, dissolution channels 7 cm wide in Yoka paleosols on dolostone may represent 140,000–4,200,000 years, and dissolution channels 3 cm wide in Biha paleosols on limestone may represent 1,000–100,000 years. The long dissolution times match extensive geological disconformities revealed by gaps in chemostratigraphic studies of these rocks [8,78].

### Paleoclimate

Gypsic and calcic horizons are today found at depths in soils proportional to mean annual precipitation [80,81]. Calcic soils are widespread in aridlands, but gypsic soils form in extreme deserts such as the Atacama Desert of Chile [214,215]. For calcic paleosols, mean annual precipitation is related to depth in the profile to calcareous nodules corrected for burial compaction from a global compilation [80], following the compaction and paleoclimatic equations in Table 1. For gypsic soils, another global compilation gives mean annual precipitation from depth to gypsum, again compaction corrected [81], using equation in Table 1. Seasonality of precipitation, defined as wettest minus driest month mean precipitation, is a function of thickness of the calcic horizon, again from a global compilation [80]. In highly seasonal climate, carbonate precipitates at a wide range of levels within soil profiles.

The calcic climofunction applied to eight Cambrian Bui paleosols in the Carrara Formation gives paleoprecipitation of 403 ± 147 mm to 636 ± 147 mm (S6 Table). Means and standard deviation for paleoprecipitation of all 8 paleosols are 515 ± 73 mm. The gypsic climofunction applied to two Ediacaran Hebinga compaction-corrected paleosols of the Stirling Quartzite gives paleoprecipitation of 267 ± 129 mm and 288 ± 129 mm (S6 Table). These Ediacaran paleosols of the Stirling Quartzite are thus hyperarid like those of the modern Atacama Desert [214,215]. The Cambrian paleosols of the Carrara Formation were semiarid to subhumid, in climate cycles that correspond with sequence stratigraphic cycles in the Carrara Formation [216].

Seasonality of precipitation, or wettest month average minus driest month average, for eight Bui paleosols range from 27 ± 22 mm to 69 ± 22 mm, which are modest, non-monsoonal seasonalities [80]. The orientation of North America during the Ediacaran and Cambrian was comparable with that of India today [97,98], where monsoonal seasonality increases with elevation of the Himalaya and Tibetan Plateau over the past 20 Ma [217]. Lack of monsoonal seasonality in the Ediacaran to Cambrian of southern California is thus evidence that the hinterland was hilly rather than mountainous. Low paleolatitude with a warm ocean to the northeast makes a summer dry season more likely than a winter-dry climate [218].

A general idea of paleotemperature can be gained from chemical index of alteration (CIA of Table 1), which is 50–55 in glacial sediments and more than 80 in tropical sediments [82]. CIA is only a very general indicator because it includes cumulative weathering through multiple episodes of redeposition. Some pedogenic paleothermometers using only forested soils [219] are less appropriate for Ediacaran and Cambrian paleosols than paleothermometers from chemical weathering (CIW) under Icelandic shrublands [83] and alkali index (AI) of North American deserts [84]. All three methods give comparable paleotemperatures (S7 Table). Ediacaran Stirling Quartzite was frigid from CIA, and has mean annual paleotemperature of 4.7 ± 4.4^o^C from AI and 10.1 ± 2.1^o^C from CIW. Cambrian paleosols of the Zabriskie Quartzite and Wood Canyon and Carrara Formations formed under temperate CIA, mean annual temperature of 9.9 ± 4.4^o^C to 13.5 ± 4.4^o^C from AI and 7.2 ± 2.1^o^C to 10.2 ± 2.1^o^C from CIW. The tropical paleolatitudes of these formations [97,98] is puzzling for such cool paleotemperatures, but there were extensive Late Ediacaran and Early Cambrian glaciations elsewhere in the world [220–223]. Paleosols formed in southern California at times of glacial expansion and relatively low sea level, not the intervening times of warmth and marine transgression.

The limestone and dolostone hosted paleosols (Biha, Pakuitah, Yoka) all have narrow ferruginized cracks most like temperate subhumid limestone weathering, rather than deep wide cracks and spongy phytokarst of tropical karst [224]. Quantitative paleoclimatic transfer functions are not yet available for paleokarst paleosols.

### Life on land

A variety of megafossils were found in paleosols of southern California, as well as within marine rocks. The non-marine nature of these fossils is indicated by their preservation in paleosol surfaces, as well as from their low boron content (S5 Table). All Ediacaran large quilted fossils were assumed to have been marine invertebrates until Seilacher [225] pointed out that they have no marine invertebrate characters, and Californian and Namibian taxa remain especially enigmatic [28]. The discoid fossil *Hallidaya brueri* [95] was abundant on the surface of Hebinga paleosols in the Stirling Quartzite both at Donna Loy Mine (Fig 11A) and both southwest and west of Johnnie. *Ernietta plateauensis* (Fig 11D-G), *Pteridinium simplex* (Fig 11D), and *Swartpuntia germsi* were found in Nataanga and Aisen profiles of the Lower Member of the Wood Canyon Formation at Donna Loy Mine (Fig 3A) and 3 miles south of Johnnie [18,35,37]. *Swartpuntia* was only found flattened on surfaces, but *Pteridinium* and *Ernietta* were partly buried in the soil (Fig 2E, 11E), or “underground” as described by Grazhdankin and Seilacher [38], although those authors probably meant sub-seafloor. The rounded bulb of *Ernietta* was buried, but the leaves protruded above the ground between ventifacted pebbles of the desert pavement (Fig 11G). The slab from 3 miles south of Johnnie (Fig 11D) with both *Pteridinium* and *Ernietta* [37] was from an area marked by a cairn and with only Aisen paleosols exposed, like paleosols with similar fossils near Donna Loy Mine (Fig 11E). Comparable modern ecosystems have been called a polsterland: scattered low mosses, liverworts, and lichens, with much bare earth exposed [137].

Some specimens of *Swartpuntia* (Fig 11B) [34] are from laminated marine shales with Cambrian trilobites of the *Fallotaspis* and *Nevadella* trilobite zones, dated at 515–518 Ma [87], and a similar form is known from very early Cambrian rocks of South Australia [226]. Thus *Swartpuntia* occasionally drifted out to sea, and did not become extinct at the end of the Cambrian like many other Ediacaran fossils [227]. Similarly in central Australia and Montana, the Ediacaran fossils *Hallidaya*, *Arumberia*, and *Noffkarkys* persist well into Cambrian paleosols until after the first appearance of trilobite trace fossils [95,228].

There are also marine fossils and rocks in these sequences distinguished by high boron content (S5 Table) and heavy YREE enrichment (Fig 10). The Cambrian bed with the trace fossil *Bergaueria* and conical fossil *Wyattia* (Fig 11F) is distinct in its REE composition (Fig 10) and shows very little internal chemical or petrographic differentiation (Fig 7). First appearance of the marine trace fossil *Manykodes pedum* [52] is taken as the base of the Cambrian, although its appearance in the stratotype section in Newfoundland is below the golden spike [87]. *Manykodes pedum* has historically been known as “*Phycodes pedum”* and “*Treptichnus pedum”* [53]. *Manykodes* does not have bundled or radiating branches like *Phycodes circinatum*, the type species of that genus [229], nor does it have long straight links like *Treptichnus bifurcus*, the type species of that genus [54,230]. Increased diversity of trace fossils in the Upper Member of the Wood Canyon Formation has been taken as an indication of the Cambrian explosion in diversity of marine life [2,4,58,231,232], but may reflect local marine transgression rather than global marine diversification and substrate revolution. Similar problems of alternating marine and freshwater habitats confound evolutionary interpretations of many Ediacaran-Cambrian successions [11,43].

Another indication of terrestrial productivity is depletion of phosphorus in the paleosols, because organic ligands are needed to mobilize this vital element for life from relatively insoluble apatite [233]. The maximal mole fraction depletion (negative tau value) of P in selected paleosols range from 7 to 55% (Table), but enrichment of P by 7% in the Bui profile (Fig 8). The red, calcareous Bui exception may be explained by phosphorus retention by pedogenic calcite and hematite, which is common in modern soils [234,235]. Phosphorus depletion in the other paleosols varies according to duration of soil development (S8 Table), as in modern soils (Batjes 2011). Phosphorus depletion as evidence of life in Ediacaran and Cambrian paleosols has been widely reported [21,93,96,232,236].

Vesicular structure in Duhubite profiles (Fig 4B) is similar to features in desert soils created when soil microbiota comes alive after a rainstorm and metabolic gases are trapped in soft mud [118]. Other indications of life on land are surface textures of *Rivularites repertus* (Fig 11I) with three features indicative of wetting, drying and growth centers: 1, chopped up or hackly appearance, 2, randomly oriented fissures that are open, partly filled, or closed with undulating sutures, and 3, microtuffets of radial growth centers [62]. Other evidence of exposure are mud cracks with mudcurls (Fig 11H) of the kind widely identified in the rock record as “Manchuriophycus”, but of debatable biological origin [237,238].

Other metrics of terrestrial productivity are proxies for soil CO_2_ levels. Modern soils have calcareous nodules and gypsum desert roses at depths within the profile proportional to measured soil CO_2_ (Table 1), which exceeds atmospheric CO_2_ because of soil respiration into restricted soil spaces [85]. This calculation is based on depths to nodules and roses reconstructed for burial compaction by 4 km using an equation in Table 1. The gypsic productivity metric applied to two Hebinga paleosols gives soil-CO_2_ of 1300 ± 552 ppm and 1451 ± 552 ppm (S6 Table). The calcic productivity metric applied to eight Bui paleosols gives 1739 ± 768 ppm soil-CO_2_–3025 ± 768 mm. Means and standard deviation for soil-CO_2_ of all eight paleosols are 2327 ± 400 ppm. These levels of paleoproductivity are similar to 1000–2000 ppm found in modern desert soils, and far short of tropical forest soils which can have as much as 104,000 ppm soil CO_2_ [85]. These new results from paleosols support inferences from clays [239] and isotopic composition [240] of marine rocks for increased biological weathering on land during the late Ediacaran.

## Comparison with Namibia

Diversity of Ediacaran fossils, paleosols, and facies of southern California are limited, but very similar to those of Namibia. Fossil vendobionts *Pteridinium*, *Ernietta*, and *Swartpuntia* known from southern California [33–35,37] were first discovered, and remain best known in Namibia [38–40,241]. Namibia and California also share *Cloudina* tubular shells in marine limestones [41,65,66]. An early record of *Pteridinium* from California [242] is now regarded as a pseudofossil [243], but new specimens of *Pteridinium simplex* (Fig 11D) from Johnnie [35,37], show convincing zigzag suture and high relief down into the matrix like Namibian specimens [38]. Also like southern California, Namibia has quartzose fluvial [45] to lagoonal sandstones [44] from a granitic source terrane of subdued relief as well as shallow marine limestones. *Pteridinium simplex* and *Ernietta plateauensis* in Namibia are also very low in boron (S5 Table), and are embedded within ferruginized weakly developed paleosols [45] similar to the Aisen pedotype of southern California. *Rangea schneiderhoehni* also was observed in a Namibian paleosol comparable with Aisen, and capped by loessic laminae [45]. This bed is within intertidal heterolithic facies with a strongly sinuous and strongly tapering paleochannel [244], a channel form known today only from tidal flats [245], and not among gutter casts below wave base as previously interpreted [344]. A reddish-brown Ediacaran paleosol with loessic cap was also seen at Pockenbank in Namibia [45] and is similar to Nataanga paleosols of southern California. *Swartpuntia germsi* from Swartpunt in Namibia was found in the same locality as *Pteridinium simplex*, which has low boron of freshwater (S5 Table), and lacks any associated paleosol features. The Swartpunt specimen of *Pteridinium* in bedded siltstone is unlike others “underground” and inflated within sandstone beds [38], and was confined to a single bedding plane. That specimen may have drifted into coastal lagoonal shales that pass upward into shales and siltstones with diversifying marine trace fossils [43]. Like other sequences showing Ediacaran-Cambrian trace fossil diversification [11], this one also is compromised by non-marine interbeds. Despite these strong biotic and paleopedological similarities, Namibia and California are not connected, or close to one another, in global plate tectonic reconstructions [97,98].

## Conclusions

Discovery of Ediacaran and Cambrian paleosols in southern California offers a new perspective on the paleoecology and biostratigraphy of Ediacaran fossils and the Cambrian explosion (Fig 12), as well as detailed information on paleoclimate and habitats (S6-S7 Table). In particular this study challenges three widely held assumptions about the habitats, diversification, and extinction of Cambrian and Ediacaran fossils.

Paleosols in the southernmost localities examined include Ediacaran-Cambrian fossils *Ernietta*, *Swartpuntia*, *Pteridinium*, and *Hallidaya* in growth position, confirming evidence from boron content (S5 Table) and YREE (Fig 10) that these vendobiont fossils grew on and within land. In the past these fossils have been assumed to have been marine [35,39,40,44,241]. This new interpretation does not extend to northern localities considered here at Westgard Pass and Mt Dunfee which include limestones with *Cloudina* and *Wyattia* [66,152], shales with *Conotubus* and *Wutubus* [48], and microbial mat structures [57]. No paleosols were observed with those fossils and their boron and other chemical content is typical of marine rocks (Table S5). Highstands of the sea also brought marine fossils including burrowing worms (*Manykodes*) and trilobites (*Olenellus*) into the southern localities. Ediacaran marine Wormworld [51] was thus ecologically distinct from terrestrial Mattressland of vendobionts [45].

This ecological distinction has implications for the idea of a Cambrian substrate revolution [57] or agronomic revolution [232], in which seafloors were little disturbed by burrowing animals during the Ediacaran but became increasingly bioturbated into the Cambrian. It is apparent from context that Grazhdankin and Seilacher’s [38] “underground Vendobionta” and Seilacher and Pflüger’s [232] “agronomic revolution” were not meant to be taken as literally terrestrial, and referred to reorganization of the sea floor in the same way as the substrate revolution of Bottjer et al. [57]. Paleosols now provide complementary evidence to assess Ediacaran-Cambrian changes on land and in the sea separately, both in Namibia and southern California. In both places Ediacaran marine shales and limestones show little evidence of bioturbation, but Cambrian *Manykodes* and then a variety of trace fossils such as *Skolithos* and *Arenicolites* reached deeper into the seafloor, as originally envisaged [57,232]. However, the new understanding is that *Ernietta*, *Pteridinium*, or *Swartpuntia* grew on land. A comparison of Ediacaran and Cambrian moderately developed paleosols does show marked increase in depth and development in California, but these sections are full of disconformities and interrupted by marine transgressions [7–8], so they cannot document whether this was rapid revolution or slow evolution of terrestrial weathering. More complete records of paleosols from South Australia show a marked rise in terrestrial productivity in the earliest Cambrian to levels higher than known during the Ediacaran or Cryogenian, but also unevenness in productivity through time [21,145]. Detailed compilations of paleosols in multiple locations will be needed to fully address the issue of what happened on land during the Cambrian explosion of life in the ocean.

The enigmatic Ediacaran vendobiont *Swartpuntia*, best known in the Ediacaran rocks [241], persisted into the Cambrian in California as late as the *Nevadella* trilobite zone at 515 Ma [33,87]. Similarly, Ediacaran soft-bodied fossils *Hallidaya*, *Arumberia*, and *Noffkarkys* ranged younger than the first appearance of trilobite trace fossils in Central Australia [95,151], and a form comparable with *Swartpunti*a was found in the earliest Cambrian rocks of South Australia [226]. This is not the generally proposed mass extinction of Ediacaran Vendobionts envisaged as sessile marine creatures ploughed up by increasing aggressive burrowing animals [43]. The view from the land provided by paleosols where vendobionts lived shows no clear mass extinction of these low diversity communities, which were unaffected by diversification of burrowing animals in the ocean.

## Supporting information

S1 Table Chemical composition (wt %) from XRF. (DOCX)

S2 Table Trace element composition (ppm) from XRF and ICP. (DOCX)

S3 Table Grain-size data from point counting thin sections (500 points). (DOCX)

S4 Table Mineral content from point counting thin sections (500 points). (DOCX)

S5 Table Potassium and boron analyses, Weaver Index (WI) of illite crystallinity, marine-threshold distance (Δ_WI_), ratio of illite/quartz (10Å/2.46Å peaks), XRD-predicted clay (%), and field lithology for selected fossils of southern California and Namibia. (DOCX)

S6 Table Interpretation of Bk metrics for Ediacaran and Cambrian paleosols of California. (DOCX)

S7 Table Paleoclimate and phosphorus depletion inferred from chemical composition of Ediacaran-Cambrian paleosols, California. (DOCX)
