# Supplementary material for: Ediacaran-Cambrian paleosols of Nevada and California
Source: PLoS One. 2025 Jun 24;20(6):e0325547. doi: 10.1371/journal.pone.0325547 (PMC12186958; doi:10.1371/journal.pone.0325547)
Supplement: S1 Table — (DOCX) [file pone.0325547.s001.docx]

**Supplementary Information for “Ediacaran-Cambrian paleosols of Nevada and California” Gregory J. Retallack***, Department of Earth Sciences, University of Oregon. Eugene, Oregon, 97403.*

**Table S1. Chemical composition (wt %) from XRF**

| Pedotype. | Sample- | SiO_2_ | TiO_2_ | Al_2_O_3_ | Fe_2_O_3_ | FeO | MnO | CaO | MgO | Na_2_O | K_2_O | P_2_O_5_ | SrO | BaO | LOI | TOTAL | g.cm^-3^ |
| --- | --- | --- | --- | --- | --- | --- | --- | --- | --- | --- | --- | --- | --- | --- | --- | --- | --- |
| sandstone | 5658 | 74.10 | 0.2 | 2.65 | 1.81 | <0.01 | 0.26 | 5.92 | 3.14 | 0.02 | 1.23 | 0.27 | 0.05 | 0.04 | 8.32 | 98.01 | 2.5968 |
| sandstone | 5659 | 74.90 | 0.2 | 2.64 | 1.85 | <0.01 | 0.26 | 6.00 | 3.29 | 0.02 | 1.22 | 0.27 | 0.05 | 0.05 | 8.32 | 99.07 | 2.6089 |
| sandstone | 5660 | 74.50 | 0.2 | 2.80 | 2.01 | 0.13 | 0.26 | 5.73 | 3.08 | 0.02 | 1.31 | 0.28 | 0.05 | 0.05 | 8.04 | 98.33 | 2.5937 |
| sandstone | 5661 | 76.10 | 0.24 | 2.79 | 1.94 | <0.01 | 0.23 | 5.41 | 2.60 | 0.01 | 1.27 | 0.31 | 0.06 | 0.04 | 7.11 | 98.11 | 2.4095 |
| sandstone | 5662 | 79.70 | 0.25 | 2.89 | 2.02 | 0.33 | 0.19 | 5.48 | 1.19 | 0.02 | 1.25 | 0.30 | 0.05 | 0.04 | 5.71 | 99.09 | 2.5920 |
| Aisen | 5663 | 90.55 | 0.51 | 2.35 | 3.55 | 0.39 | <0.01 | 0.25 | 0.15 | 0.04 | 1.18 | 0.044 | <0.01 | 0.02 | 0.84 | 99.48 | 2.8933 |
| Aisen | 5664 | 91.14 | 0.57 | 1.94 | 3.87 | 0.59 | <0.01 | 0.19 | 0.15 | 0.04 | 0.94 | 0.032 | 0.01 | 0.02 | 0.86 | 99.76 | 2.7755 |
| Aisen | 5665 | 93.16 | 0.5 | 1.29 | 2.93 | 0.66 | <0.01 | 0.13 | 0.12 | 0.03 | 0.61 | 0.03 | 0.01 | 0.01 | 0.74 | 99.86 | 2.5991 |
| Hebinga | 5351 | 92.1 | 0.38 | 2.98 | 1.48 | 1.15 | 0.01 | 0.08 | 0.19 | 0.05 | 1.61 | 0.02 | <0.01 | 0.03 | 0.29 | 99.22 | 2.6602 |
| Hebinga | 5352 | 92.20 | 0.58 | 2.17 | 2.53 | 2.44 | 0.02 | 0.06 | 0.08 | 0.05 | 1.29 | 0.01 | <0.01 | 0.02 | -0.46 | 98.55 | 2.6371 |
| Hebinga | 5353 | 91.50 | 0.65 | 2.76 | 1.88 | 1.54 | 0.02 | 0.05 | 0.12 | 0.04 | 1.55 | 0.01 | <0.01 | 0.03 | 0.02 | 98.63 | 2.6454 |
| Hebinga | 5354 | 93.20 | 0.61 | 3.07 | 1.23 | 0.96 | 0.01 | 0.04 | 0.20 | 0.05 | 1.63 | 0.02 | <0.01 | 0.03 | 0.10 | 101.19 | 2.6707 |
| Hebinga | 5355 | 91.50 | 0.57 | 3.40 | 2.81 | 2.56 | 0.02 | 0.03 | 0.19 | 0.05 | 1.75 | 0.009 | <0.01 | 0.03 | -0.43 | 100.52 | 2.6543 |
| Aisen | fossil | 60.90 | 0.23 | 3.57 | 4.65 | 1.77 | 0.21 | 10.65 | 4.75 | 0.04 | 1.68 | 0.13 | 0.01 | 0.03 | 13.45 | 100.3 | 2.5920 |
| Nataanga | 5356 | 76.70 | 0.24 | 3.94 | 6.39 | 2.74 | 0.21 | 6.14 | 1.24 | 0.05 | 1.96 | 0.13 | 0.01 | 0.04 | 3.09 | 100.14 | 2.3526 |
| Nataanga | 5357 | 83.20 | 0.31 | 4.13 | 7.43 | 4.75 | 0.17 | 2.91 | 0.28 | 0.05 | 2.04 | 0.13 | <0.01 | 0.03 | 0.74 | 101.42 | 2.2577 |
| Nataanga | 5358 | 86.80 | 0.33 | 4.71 | 4.08 | 2.84 | 0.11 | 1.67 | 0.30 | 0.05 | 2.26 | 0.15 | <0.01 | 0.04 | 1.14 | 101.64 | 2.3312 |
| Bui | 5832 | 56.26 | 0.78 | 21.59 | 6.24 | 1.67 | 0.01 | 0.22 | 2.16 | 0.34 | 7.32 | 0.123 | <0.01 | 0.07 | 4.22 | 99.34 | 2.6882 |
| Bui | 5833 | 50.16 | 0.8 | 21.94 | 8.10 | 1.17 | 0.04 | 1.33 | 2.78 | 0.26 | 7.76 | 0.131 | <0.01 | 0.07 | 6.21 | 99.59 | 2.6644 |
| Bui | 5834 | 52.64 | 0.83 | 21.56 | 8.91 | 1.29 | 0.01 | 0.36 | 2.06 | 0.37 | 7.55 | 0.131 | <0.01 | 0.06 | 4.51 | 99.00 | 2.7381 |
| Bui | 5835 | 52.29 | 0.81 | 21.52 | 8.86 | 1.29 | 0.02 | 0.58 | 2.26 | 0.35 | 7.49 | 0.141 | <0.01 | 0.06 | 4.88 | 99.27 | 2.7255 |
| Aingebite | 5836 | 52.99 | 0.73 | 19.86 | 8.56 | 2.97 | 0.03 | 1.03 | 3.48 | 0.34 | 6.17 | 0.139 | <0.01 | 0.13 | 5.58 | 99.05 | 2.6279 |
| Aingebite | 5837 | 51.17 | 0.81 | 22.09 | 8.23 | 1.67 | 0.03 | 0.90 | 2.63 | 0.29 | 7.65 | 0.14 | <0.01 | 0.17 | 5.58 | 99.71 | 2.7808 |
| Aingebite | 5838 | 43.92 | 0.65 | 20.33 | 8.92 | 1.03 | 0.08 | 3.65 | 4.24 | 0.12 | 7.22 | 0.11 | <0.01 | 0.07 | 9.87 | 99.19 | 2.7122 |
| Pohonta | 5839 | 47.66 | 0.78 | 21.84 | 8.26 | 1.16 | 0.05 | 2.08 | 3.27 | 0.24 | 7.78 | 0.145 | <0.01 | 0.07 | 7.48 | 99.66 | 2.7245 |
| Pohonta | 5840 | 48.49 | 0.79 | 22.11 | 8.42 | 1.16 | 0.04 | 1.7 | 3.07 | 0.26 | 7.82 | 0.149 | <0.01 | 0.06 | 6.89 | 99.82 | 2.7568 |
| Pohonta | 5841 | 47.54 | 0.79 | 21.27 | 8.02 | 1.16 | 0.06 | 2.36 | 3.44 | 0.24 | 7.57 | 0.138 | <0.01 | 0.06 | 7.85 | 99.35 | 2.7164 |
| Pohonta | 5842 | 48.95 | 0.79 | 20.84 | 8.32 | 1.29 | 0.04 | 1.9 | 3.21 | 0.3 | 7.27 | 0.136 | <0.01 | 0.13 | 7.08 | 98.98 | 2.7142 |
| Wookki | 5843 | 52.43 | 0.7 | 19.84 | 10.11 | 4.11 | 0.03 | 0.83 | 3.97 | 0.28 | 5.82 | 0.138 | <0.01 | 0.06 | 5.51 | 99.73 | 2.7134 |
| Wookki | 5844 | 45.77 | 0.71 | 18.59 | 7.64 | 1.56 | 0.12 | 4.22 | 4.74 | 0.17 | 6.38 | 0.107 | <0.01 | 0.07 | 10.45 | 98.98 | 2.6605 |
| Bisapi | 5798 | 94.27 | 0.07 | 1.51 | 0.97 | 0.51 | 0.01 | 0.53 | 0.18 | 0.02 | 0.45 | 0.017 | 0.01 | 0.63 | 0.66 | 99.33 | 2.6770 |
| Bisapi | 5799 | 96.96 | 0.05 | 0.94 | 0.66 | 0.52 | <0.01 | 0.22 | 0.10 | 0.01 | 0.28 | 0.014 | <0.01 | 0.03 | 0.31 | 99.57 | 2.5987 |
| Bisapi | 5800 | 97.58 | 0.04 | 0.69 | 0.82 | 0.65 | 0.01 | 0.06 | 0.06 | 0.007 | 0.19 | 0.012 | <0.01 | 0.02 | 0.07 | 99.55 | 2.5715 |
| Bisapi | 5801 | 97.2 | 0.06 | 0.85 | 0.69 | 0.64 | <0.01 | 0.05 | 0.06 | 0.007 | 0.24 | 0.01 | <0.01 | <0.01 | 0.09 | 99.21 | 2.6554 |
| error | all | 2.705 | 0.06 | 0.825 | 0.395 |  | 0.22 | 0.18 | 0.11 | 0.13 | 0.025 | 0.030 |  |  |  | 0.35 | 0.009 |

Note: Samples are all R-numbers but “fossil” is specimen F123719A in the Condon Collection, University of Oregon.. Errors are from 10 replicate analyses of the standard, CANMET SDMS2 (British Columbia granodioritic sand).
