# Supplementary material for: Ediacaran-Cambrian paleosols of Nevada and California
Source: PLoS One. 2025 Jun 24;20(6):e0325547. doi: 10.1371/journal.pone.0325547 (PMC12186958; doi:10.1371/journal.pone.0325547)
Supplement: S2 Table — (DOCX) [file pone.0325547.s002.docx]

**Supplementary Information for “Ediacaran-Cambrian paleosols of Nevada and California” Gregory J. Retallack***, Department of Earth Sciences, University of Oregon. Eugene, Oregon, 97403.*

Table S2. Trace element composition (ppm) from XRF and ICP.

| pedotype | specimen | Ba | Ce | Cr | Cs | Dy | Er | Eu | Ga | Gd | Hf | Ho | La | Lu | Nb |
| --- | --- | --- | --- | --- | --- | --- | --- | --- | --- | --- | --- | --- | --- | --- | --- |
| sandstone | 5658 | 412 | 51.1 | 20 | 0.46 | 5.34 | 3.16 | 1.27 | 2.9 | 5.96 | 8.9 | 1.09 | 16.5 | 0.46 | 4.3 |
| sandstone | 5659 | 454 | 52.2 | 20 | 0.45 | 5.41 | 3.34 | 1.28 | 2.6 | 6.43 | 8.1 | 1.18 | 17.1 | 0.46 | 3.8 |
| sandstone | 5660 | 449 | 51.9 | 20 | 0.53 | 5.55 | 3.21 | 1.36 | 3.1 | 6.52 | 8.1 | 1.23 | 16.3 | 0.44 | 3.8 |
| sandstone | 5661 | 432 | 58.6 | 30 | 0.5 | 6.04 | 3.69 | 1.43 | 3 | 7.39 | 9.7 | 1.31 | 19.3 | 0.51 | 4.7 |
| sandstone | 5662 | 420 | 57.3 | 30 | 0.62 | 5.7 | 3.31 | 1.45 | 3.1 | 7.24 | 10.2 | 1.24 | 18.8 | 0.51 | 4.7 |
| Hebinga | 5351 | 216 | 33.5 | 20 | 0.44 | 1.47 | 0.75 | 0.32 | 3.4 | 1.34 | 2.3 | 0.27 | 17.4 | 0.09 | 2.6 |
| Hebinga | 5352 | 206 | 17.9 | 20 | 0.37 | 1.08 | 0.63 | 0.26 | 2.3 | 1.00 | 3.7 | 0.22 | 9.3 | 0.12 | 3.2 |
| Hebinga | 5353 | 209 | 27.8 | 20 | 0.41 | 1.28 | 0.68 | 0.36 | 3.2 | 1.44 | 3.1 | 0.25 | 13.6 | 0.1 | 3.6 |
| Hebinga | 5354 | 217 | 74.2 | 20 | 0.49 | 2.6 | 1.39 | 0.64 | 3.7 | 2.68 | 6.2 | 0.47 | 37.5 | 0.19 | 8.5 |
| Hebinga | 5355 | 210 | 24.7 | 20 | 0.49 | 1.67 | 0.99 | 0.34 | 3.9 | 1.54 | 3.6 | 0.34 | 13.5 | 0.14 | 6.0 |
| Aisen | fossil | 241 | 23.8 | 20 | 0.59 | 1.5 | 0.92 | 0.41 | 5 | 1.67 | 3.1 | 0.3 | 10.9 | 0.13 | 2.7 |
| Nataanga | 5356 | 339 | 25.9 | 30 | 0.72 | 1.49 | 0.85 | 0.37 | 5.4 | 1.75 | 3 | 0.28 | 11.8 | 0.14 | 2.7 |
| Nataanga | 5357 | 282 | 26.4 | 30 | 0.77 | 1.79 | 1 | 0.46 | 5.9 | 1.81 | 4 | 0.35 | 12 | 0.15 | 3.5 |
| Nataanga | 5358 | 300 | 32.6 | 20 | 0.92 | 2.23 | 1.26 | 0.5 | 6.2 | 2.45 | 4.2 | 0.44 | 14.9 | 0.17 | 3.8 |
| error | all | 0.1 | 0.1 | 0.21 | 0.25 | 0.05 | 0.03 | 0.1 | 0.05 | 0.01 | 0.2 | 0.01 | 0.1 | 0.01 | 0.2 |

| pedotype | specimen | Nd | Pr | Rb | Sm | Sn | Sr | Ta | Tb | Th | Tm | U | V | W | Y | Yb | Zr |
| --- | --- | --- | --- | --- | --- | --- | --- | --- | --- | --- | --- | --- | --- | --- | --- | --- | --- |
| sandstone | 5658 | 24.2 | 5.31 | 31.7 | 6.51 | <1 | 399 | 0.4 | 0.91 | 5.11 | 0.43 | 2.54 | 18 | 1 | 30.2 | 3.18 | 367 |
| sandstone | 5659 | 24.3 | 5.41 | 31.5 | 6.36 | <1 | 434 | 0.4 | 1.04 | 4.98 | 0.44 | 2.59 | 20 | 1 | 30.9 | 3.09 | 349 |
| sandstone | 5660 | 25.9 | 5.47 | 33.2 | 7.14 | <1 | 401 | 0.3 | 0.97 | 5.05 | 0.48 | 2.23 | 18 | 1 | 31.2 | 3.28 | 342 |
| sandstone | 5661 | 28.2 | 6.2 | 32.8 | 7.92 | <1 | 547 | 0.5 | 1.1 | 6.75 | 0.45 | 2.96 | 21 | 1 | 34.3 | 3.27 | 388 |
| sandstone | 5662 | 27.6 | 5.92 | 31.8 | 7.15 | <1 | 442 | 0.4 | 1.12 | 5.68 | 0.51 | 2.22 | 21 | 1 | 33.9 | 3.13 | 431 |
| Hebinga | 5351 | 12.4 | 3.61 | 34.4 | 1.6 | <1 | 26.5 | 0.2 | 0.24 | 3.72 | 0.11 | 0.84 | 22 | 2 | 8.1 | 0.71 | 79 |
| Hebinga | 5352 | 7.1 | 2.04 | 25.9 | 1.15 | <1 | 26.6 | 0.2 | 0.16 | 3.84 | 0.08 | 0.75 | 16 | 2 | 6.1 | 0.57 | 130 |
| Hebinga | 5353 | 11.9 | 3.28 | 31.8 | 2.26 | <1 | 22.8 | 0.3 | 0.21 | 4.42 | 0.12 | 0.84 | 23 | 2 | 7.2 | 0.69 | 112 |
| Hebinga | 5354 | 28.4 | 8.36 | 35.6 | 4.5 | 1 | 22.7 | 0.6 | 0.38 | 8.95 | 0.18 | 1.71 | 36 | 5 | 13.5 | 1.32 | 222 |
| Hebinga | 5355 | 9.3 | 2.73 | 37 | 1.68 | 1 | 18.8 | 0.4 | 0.27 | 4.75 | 0.14 | 1.11 | 32 | 5 | 9.5 | 0.91 | 128 |
| Aisen | fossil | 10.2 | 2.75 | 38 | 2.02 | 1 | 84.9 | 0.2 | 0.27 | 2.33 | 0.13 | 1.58 | 22 | 1 | 9 | 0.85 | 111 |
| Nataanga | 5356 | 11.0 | 2.95 | 43.8 | 2.04 | 1 | 97.9 | 0.2 | 0.25 | 2.46 | 0.13 | 2.3 | 17 | 2 | 8.3 | 0.81 | 109 |
| Nataanga | 5357 | 11.5 | 3.12 | 45.2 | 2.38 | 1 | 65.2 | 0.2 | 0.29 | 2.9 | 0.15 | 1.57 | 14 | 1 | 9.9 | 0.83 | 146 |
| Nataanga | 5358 | 14.3 | 3.76 | 51.5 | 2.64 | 2 | 34.8 | 0.2 | 0.37 | 3.43 | 0.17 | 1.46 | 15 | 1 | 13 | 1.12 | 152 |
| error | all | 0.1 | 0.03 | 0.2 | 0.05 | 1 | 0.1 | 0.1 | 0.01 | 0.05 | 0/01 | 0.05 | 5 | 1 | 0.02 | 0.05 | 2 |

Note: Samples are all R-numbers but “fossil” is specimen F123719A in the Condon Collection, University of Oregon
