# Supplementary material for: Ediacaran-Cambrian paleosols of Nevada and California
Source: PLoS One. 2025 Jun 24;20(6):e0325547. doi: 10.1371/journal.pone.0325547 (PMC12186958; doi:10.1371/journal.pone.0325547)
Supplement: S3 Table — (DOCX) [file pone.0325547.s003.docx]

**Supplementary Information for “Ediacaran-Cambrian paleosols of Nevada and California” Gregory J. Retallack***, Department of Earth Sciences, University of Oregon. Eugene, Oregon, 97403.*

Table S3. Grain-size data from point counting thin sections (500 points).

| Pedotype | Hoz | No. | % gravel | % sand | % silt | % clay | Textural class | Grain fabric | Plasmic fabric |
| --- | --- | --- | --- | --- | --- | --- | --- | --- | --- |
| Hebinga | A | R5351 | 6.2 | 43.8 | 31.6 | 18.4 | loam | granular | silasepic |
| Hebinga | By | R5352 | 8.6 | 43.2 | 30.6 | 17.6 | loam | granular | silasepic |
| Hebinga | By | R5353 | 6.8 | 47.8 | 30.4 | 15.0 | loam | granular | silasepic |
| Hebinga | C | R5354 | 11.2 | 44.0 | 31.0 | 13.8 | sandy loam | granular | silasepic |
| Hebinga | C | R5355 | 5.8 | 48.4 | 33.4 | 12.4 | sandy loam | granular | silasepic |
| Nataanga | A | R5456 | 0 | 33.6 | 46.8 | 19.6 | loam | granular | silasepic |
| Nataanga | C | R5357 | 0 | 29.8 | 51.6 | 18.6 | silt loam | granular | silasepic |
| Nataanga | C | R5358 | 0 | 31.4 | 48.4 | 20.2 | loam | intertextic | insepic |
| Nataanga | C | R5359 | 0 | 30.6 | 51.0 | 18.4 | silt loam | intertextic | calciasepic |
| sandstone | top | R5658 | 0 | 26.8 | 53.6 | 19.6 | silt loam | intertextic | silasepic |
| sandstone | middle | R5659 | 0 | 27.6 | 53.2 | 19.2 | silt loam | intertextic | silasepic |
| sandstone | middle | R5660 | 0 | 27.8 | 53.4 | 18.8 | silt loam | intertextic | silasepic |
| sandstone | middle | R5661 | 0 | 27.4 | 54.4 | 18.2 | silt loam | granular | silasepic |
| sandstone | bottom | R5662 | 0 | 25.4 | 57.0 | 17.6 | silt loam | granular | silasepic |
| Aisen | top | R5663 | 1.8 | 39.6 | 39.0 | 19.6 | loam | granular | silasepic |
| Aisen | middle | R5664 | 3.4 | 41.4 | 37.6 | 17.2 | loam | granular | silasepic |
| Aisen | bottom | R5665 | 3.0 | 44.0 | 35.0 | 18.0 | loam | granular | silasepic |
| Bisapi | above | R5797 | 2.6 | 59.6 | 30.0 | 7.8 | sandy loam | granular | silasepic |
| Bisapi | A | R5798 | 1.4 | 49.8 | 36.0 | 12.8 | loam | intertextic | silasepic |
| Bisapi | Bw | R5799 | 0 | 60.4 | 23.6 | 16.0 | sandy loam | intertextic | silasepic |
| Bisapi | Bw | R5800 | 1.8 | 69.8 | 12.8 | 15.6 | sandy loam | intertextic | silasepic |
| Bisapi | Bw | R5801 | 0.6 | 66.8 | 25.0 | 7.6 | sandy loam | intertextic | silasepic |
| Bisapi | C | R5802 | 1.2 | 64.6 | 27.4 | 6.8 | sandy loam | intertextic | silasepic |
| Bisapi | C | R5803 | 0.4 | 67.8 | 24.8 | 6.8 | sandy loam | intertextic | silasepic |
| Buinga | above | R5804 | 5.4 | 69.4 | 19.8 | 5.6 | sandy loam | granular | silasepic |
| Buinga | A | R5805 | 0.6 | 52.6 | 29.2 | 17.6 | sandy loam | granular | silasepic |
| Buinga | A | R5806 | 0.8 | 51.8 | 29.6 | 17.8 | loam | granular | silasepic |
| Buinga | Bg | R5807 | 1.0 | 45.4 | 35.8 | 17.8 | loam | granular | silasepic |
| Buinga | Bg | R5808 | 0.6 | 61.8 | 27.8 | 9.8 | sandy loam | granular | silasepic |
| Buinga | C | R5809 | 1.6 | 86.6 | 10.0 | 1.8 | sand | granular | silasepic |
| Buinga | C | R5810 | 1.2 | 68.2 | 23.4 | 7.2 | sandy loam | granular | silasepic |
| Paattsi | above | R5811 | 0 | 34.8 | 35.4 | 29.8 | clay loam | agglomeroplasmic | mosepic |
| Paattsi | A | R5812 | 1.4 | 49.0 | 32.6 | 17.0 | loam | intertextic | silasepic |
| Paattsi | C | R5813 | 0 | 63.2 | 18.4 | 18.4 | sandy loam | intertextic | silasepic |
| Paattsi | C | R5814 | 0.6 | 66.4 | 21.8 | 11.0 | sandy loam | intertextic | silasepic |
| Pakuitah | above | R5815 | 1.6 | 46.4 | 34.8 | 17.2 | loam | intertextic | silasepic |
| Pakuitah | A | R5816 | 15.6 | 41.6 | 36.2 | 6.6 | silt loam | agglomeroplasmic | calciasepic |
| Pakuitah | A | R5817 | 8.2 | 39.2 | 34.8 | 17.8 | loam | intertextic | calciasepic |
| Pakuitah | C | R5818 | 2.2 | 37.4 | 44.4 | 16.0 | loam | agglomeroplasmic | calciasepic |
| Pakuitah | C | R5819 | 3.0 | 40.6 | 39.6 | 16.8 | loam | agglomeroplasmic | calciasepic |
| Oompin | above | R5820 | 1.8 | 26.4 | 48.0 | 23.6 | loam | intertextic | calciasepic |
| Oompin | A | R5821 | 0.6 | 60.0 | 25.8 | 13.6 | sandy loam | agglomeroplasmic | calciasepic |
| Oompin | C | R5822 | 0.8 | 65.4 | 23.6 | 10.6 | sandy loam | porphyroskelic | calciasepic |
| Bui | above | R5831 | 0 | 4.2 | 79.8 | 16.0 | silt loam | intertextic | silasepic |
| Bui | A | R5832 | 0 | 9.4 | 59.0 | 31.6 | silty clay loam | porphyroskelic | clinobimaspic |
| Bui | A | R5833 | 0 | 1.6 | 73.4 | 25.0 | silt loam | agglomeroplasmic | skelmosepic |
| Bui | Bk | R5834 | 0 | 5.4 | 71.4 | 23.2 | silt loam | agglomeroplasmic | skelmosepic |
| Bui | Bk | R5835 | 0 | 8.2 | 70.6 | 21.2 | silt loam | agglomeroplasmic | insepic |
| Aingebite | A | R5836 | 0 | 6.2 | 64.8 | 29.0 | silty clay loam | agglomeroplasmic | clinobimasepic |
| Aingebite | C | R5837 | 0 | 8.4 | 63.6 | 28.0 | silty clay loam | intertextic | insepic |
| Pohonta | A | R5838 | 0 | 14.4 | 57.2 | 28.4 | silty clay loam | intertextic | argillasepic |
| Pohonta | Bw | R5839 | 0 | 6.6 | 62.2 | 31.2 | silty clay loam | porphyroskelic | mosepic |
| Pohonta | Bw | R5840 | 0 | 8.2 | 61.6 | 30.2 | silty clay loam | porphyroskelic | skelmospic |
| Pohonta | Bw | R5841 | 0 | 3.4 | 69.0 | 27.6 | silty clay loam | porphyroskelic | skelmospic |
| Pohonta | C | R5842 | 5.8 | 17.6 | 47.6 | 29.0 | silty clay loam | agglomeroplasmic | insepic |
| Wookki | A | R5843 | 5.0 | 20.0 | 44.2 | 30.8 | silty clay loam | porphyroskelic | mosepic |
| Wookki | C | R5844 | 0 | 3.4 | 72.4 | 24.2 | silt loam | intertextic | argillasepic |
| Duhubite | A | R5851 | 0.4 | 42.0 | 39.8 | 17.8 | loam | intertextic | silasepic |
| Duhubite | A | R5852 | 1.0 | 41.4 | 43.4 | 14.2 | loam | intertextic | silasepic |
| Duhubite | C | R5853 | 1.0 | 57.6 | 35.0 | 6.4 | sandy loam | granular | silasepic |
| Duhubite | C | R5854 | 1.8 | 66.4 | 28.2 | 3.6 | sandy loam | granular | silasepic |
