# Supplementary material for: Ediacaran-Cambrian paleosols of Nevada and California
Source: PLoS One. 2025 Jun 24;20(6):e0325547. doi: 10.1371/journal.pone.0325547 (PMC12186958; doi:10.1371/journal.pone.0325547)
Supplement: S4 Table — (DOCX) [file pone.0325547.s004.docx]

**Supplementary Information for “Ediacaran-Cambrian paleosols of Nevada and California” Gregory J. Retallack***, Dept Earth Sci. Univ. Oregon, Eugene, OR 97403.*

Table S4. Mineral content from point counting thin sections (500 points)

| Pedotype | Hoz | No. | % clay | % gypsum | % micrite | % sparry calcite | % shell calcite | % ooids | % dolomite rhombs | % rock | % feldspar | % mica | % quartz | % opaque |
| --- | --- | --- | --- | --- | --- | --- | --- | --- | --- | --- | --- | --- | --- | --- |
| Hebinga | A | R5351 | 19.4 | 0 | 0 | 0 | 0 | 0 | 0 | 6.2 | 37.8 | 1.0 | 34.4 | 1.2 |
| Hebinga | By | R5352 | 18.4 | 0 | 0 | 0 | 0 | 0 | 0 | 3.0 | 37.8 | 1.0 | 35.6 | 4.2 |
| Hebinga | By | R5353 | 16.2 | 0 | 0 | 0 | 0 | 0 | 0 | 5.0 | 34.4 | 3.8 | 37.4 | 3.2 |
| Hebinga | C | R5354 | 15.2 | 3.0 | 0 | 0 | 0 | 0 | 0 | 2.6 | 34.6 | 1.6 | 39.8 | 3.2 |
| Hebinga | C | R5355 | 20.4 | 1.6 | 0 | 0 | 0 | 0 | 0 | 2,2 | 34.6 | 0.8 | 36.2 | 4.2 |
| Nataanga | C | R5456 | 21.4 | 0 | 0 | 0 | 0 | 0 | 5.8 | 2.0 | 30.4 | 7.6 | 27.2 | 5.6 |
| Nataanga | C | R5357 | 19.8 | 0 | 0 | 0 | 0 | 0 | 4.6 | 4.0 | 32.4 | 4.2 | 30.4 | 4.6 |
| Nataanga | C | R5358 | 20.4 | 0 | 0 | 0 | 0 | 0 | 4.2 | 4.4 | 31.4 | 8.2 | 27.8 | 3.6 |
| Nataanga | C | R5359 | 19.4 | 0 | 0 | 0 | 0 | 0 | 13.6 | 6.0 | 29.2 | 5.2 | 23.6 | 3.0 |
| sandstone | top | R5658 | 21.8 | 0 | 0 | 0 | 0 | 0 | 1.0 | 2.4 | 31.6 | 5.6 | 35.2 | 2.4 |
| sandstone | middle | R5659 | 22.4 | 0 | 0 | 0 | 0 | 0 | 0 | 1.8 | 34.8 | 8.0 | 29.6 | 3.4 |
| sandstone | middle | R5660 | 23.8 | 0 | 0 | 0 | 0 | 0 | 0 | 4.6 | 34.4 | 3.4 | 29.8 | 4.0 |
| sandstone | middle | R5661 | 25.4 | 0 | 0 | 0 | 0 | 0 | 0 | 3.8 | 32.0 | 3.6 | 32.0 | 3.2 |
| sandstone | bottom | R5662 | 20.0 | 0 | 0 | 0 | 0 | 0 | 0 | 4.6 | 33.6 | 5.4 | 33.2 | 3.2 |
| Aisen | top | R5663 | 20.6 | 0 | 0 | 0 | 0 | 0 | 1.4 | 2.2 | 33.0 | 4.8 | 35.4 | 2.6 |
| Aisen | middle | R5664 | 21.4 | 0 | 0 | 0 | 0 | 0 | 0 | 1.8 | 31.4 | 3.8 | 28.6 | 3.0 |
| Aisen | bottom | R5665 | 22.2 | 0 | 0 | 0 | 0 | 0 | 0 | 1.6 | 31.0 | 2.8 | 37.6 | 4.8 |
| Bisapi | above | R5797 | 10.6 | 0 | 0 | 1.0 | 0 | 0 | 0 | 1.8 | 40.8 | 2.6 | 42.0 | 1.2 |
| Bisapi | A | R5798 | 16.0 | 0 | 0 | 1.2 | 0 | 0 | 0 | 1.0 | 44.4 | 0.2 | 36.2 | 1.0 |
| Bisapi | Bw | R5799 | 16.8 | 0 | 0 | 0 | 0 | 0 | 0 | 1.2 | 38.2 | 2.6 | 38.2 | 3.0 |
| Bisapi | Bw | R5800 | 16.4 | 0 | 0 | 0 | 0 | 0 | 0 | 2.6 | 39.6 | 2.0 | 36.6 | 2.8 |
| Bisapi | Bw | R5801 | 8.8 | 0 | 0 | 0 | 0 | 0 | 0 | 8.0 | 39.2 | 0.8 | 41.0 | 2.2 |
| Bisapi | C | R5802 | 7.8 | 0 | 0 | 0 | 0 | 0 | 0 | 3.2 | 45.8 | 1.0 | 39.2 | 3.0 |
| Bisapi | C | R5803 | 4.8 | 0 | 0 | 0 | 0 | 0 | 0 | 5.0 | 44.0 | 2.8 | 40.8 | 2.6 |
| Buinga | above | R5804 | 8.2 | 0 | 0 | 1.2 | 0 | 0 | 0 | 2.2 | 27.2 | 0.2 | 59.8 | 1.0 |
| Buinga | A | R5805 | 18.0 | 0 | 0 | 0.4 | 0 | 0 | 0 | 3.0 | 31.6 | 0.4 | 45.0 | 1.6 |
| Buinga | A | R5806 | 19.2 | 0 | 0 | 1.0 | 0 | 0 | 0 | 4.2 | 35.0 | 0.6 | 38.4 | 1.6 |
| Buinga | Bg | R5807 | 20.8 | 0 | 0 | 0.8 | 0 | 0 | 0 | 3.8 | 36.8 | 1.4 | 34.4 | 2.0 |
| Buinga | Bg | R5808 | 11.4 | 0 | 0 | 0.4 | 0 | 0 | 0 | 3.2 | 34.4 | 0.2 | 49.4 | 1.0 |
| Buinga | C | R5809 | 2.2 | 0 | 0 | 0 | 0 | 0 | 0 | 8.2 | 42.0 | 0.6 | 45.4 | 1.6 |
| Buinga | C | R5810 | 8.2 | 0 | 0 | 0 | 0 | 0 | 0 | 7.0 | 37.6 | 1.6 | 42.0 | 3.4 |
| Paattsi | above | R5811 | 31.6 | 0 | 0 | 0 | 0 | 0 | 0 | 0.8 | 30.2 | 9.8 | 26.2 | 1.4 |
| Paattsi | A | R5812 | 21.8 | 0 | 0 | 0.4 | 0 | 0 | 0 | 1.0 | 35.6 | 2.2 | 37.0 | 2.0 |
| Paattsi | C | R5813 | 18.8 | 0 | 0 | 0 | 0 | 0 | 0 | 1.0 | 35.0 | 0.6 | 42.6 | 2.0 |
| Paattsi | C | R5814 | 14.6 | 0 | 0 | 0 | 0 | 0 | 0 | 1.8 | 35.8 | 1.2 | 44.2 | 2.4 |
| Pakuitah | above | R5815 | 19.8 | 0 | 0 | 0 | 0 | 0 | 0 | 0.8 | 34.6 | 3.4 | 37.6 | 3.8 |
| Pakuitah | A | R5816 | 7.6 | 0.6 | 36.6 | 8.0 | 22.0 | 5.0 | 0 | 1.8 | 6.0 | 1.4 | 4.6 | 6.4 |
| Pakuitah | A | R5817 | 20.8 | 0.8 | 4.6 | 14.0 | 20.8 | 0.2 | 0 | 6.2 | 11.4 | 2.8 | 11.8 | 6.6 |
| Pakuitah | C | R5818 | 22.0 | 0.6 | 6.6 | 18.2 | 8.8 | 0 | 0 | 6.2 | 16.0 | 2.6 | 15.2 | 3.8 |
| Pakuitah | C | R5819 | 17.6 | 0 | 17.6 | 3.0 | 7.0 | 0 | 0 | 8.0 | 18.0 | 0 | 20.0 | 3.0 |
| Oompin | above | R5820 | 25.4 | 0 | 0 | 8.4 | 6.4 | 0.4 | 0 | 7.6 | 22.6 | 3.6 | 23.8 | 1.8 |
| Oompin | A | R5821 | 15.8 | 0.8 | 10.2 | 19.2 | 16.6 | 0 | 0 | 2.8 | 16.8 | 1.4 | 14.4 | 2.0 |
| Oompin | C | R5822 | 11.2 | 2.4 | 5.6 | 30.8 | 16.6 | 0 | 0 | 10.4 | 10.0 | 0 | 10.0 | 3.0 |
| Bui | above | R5831 | 19.4 | 0 | 0 | 0 | 0 | 0 | 0 | 0.6 | 36.8 | 14.8 | 27.4 | 1.0 |
| Bui | A | R5832 | 30.0 | 0 | 0 | 0 | 0 | 0 | 0 | 0.8 | 33.6 | 7.0 | 27.6 | 1.0 |
| Bui | A | R5833 | 24.6 | 0 | 0 | 0 | 0 | 0 | 0 | 3.0 | 34.4 | 5.2 | 30.2 | 2.6 |
| Bui | Bk | R5834 | 22.8 | 0 | 6.6 | 0 | 0 | 0 | 0 | 0.6 | 33.4 | 5.8 | 26.0 | 4.8 |
| Bui | Bk | R5835 | 22.8 | 0 | 2.0 | 0 | 0 | 0 | 0 | 0.6 | 37.4 | 8.2 | 27.4 | 1.6 |
| Aingebite | A | R5836 | 28.6 | 0 | 1.2 | 0 | 0 | 0 | 0 | 1.6 | 33.6 | 5.6 | 27.4 | 2.0 |
| Aingebite | C | R5837 | 27.8 | 0 | 0 | 0 | 0 | 0 | 0 | 1.0 | 32.8 | 6.8 | 28.4 | 3.2 |
| Pohonta | A | R5838 | 30.0 | 0 | 0 | 0 | 0 | 0 | 0 | 2.0 | 32.0 | 6.8 | 25.6 | 3.6 |
| Pohonta | Bw | R5839 | 30.0 | 0 | 0 | 0 | 0 | 0 | 0 | 2.2 | 30.2 | 6.4 | 25.0 | 6.0 |
| Pohonta | Bw | R5840 | 30.2 | 0 | 2.8 | 0 | 0 | 0 | 0 | 0.8 | 31.8 | 6.4 | 24.8 | 3.2 |
| Pohonta | Bw | R5841 | 27.0 | 0 | 1.4 | 0 | 0 | 0 | 0 | 1.0 | 32.4 | 5.4 | 28.2 | 4.6 |
| Pohonta | C | R5842 | 28.2 | 0 | 0.4 | 0 | 0 | 0 | 0 | 1.0 | 32.6 | 6.0 | 29.2 | 2.6 |
| Wookki | A | R5843 | 33.0 | 0 | 0 | 0 | 0 | 0 | 0 | 4.2 | 31.6 | 3.4 | 26.4 | 1.4 |
| Wookki | C | R5844 | 24.4 | 0 | 0 | 0 | 0 | 0 | 12.0 | 1.4 | 36.4 | 4.2 | 20.1 | 1.4 |
| Duhubite | A | R5851 | 18.0 | 0 | 0 | 0 | 0 | 0 | 20.6 | 1.2 | 30.4 | 0.6 | 26.2 | 3.0 |
| Duhubite | A | R5852 | 15.6 | 0 | 0 | 0 | 0 | 0 | 19.4 | 1.4 | 34.8 | 1.0 | 25.4 | 2.6 |
| Duhubite | C | R5853 | 7.2 | 0 | 0 | 0 | 0 | 0 | 13.2 | 0.4 | 34.0 | 1.8 | 42.2 | 1.2 |
| Duhubite | C | R5854 | 3.6 | 0 | 0 | 0 | 0 | 0 | 0 | 2.8 | 41.0 | 3.4 | 46.4 | 2.8 |
