# Supplementary material for: Ediacaran-Cambrian paleosols of Nevada and California
Source: PLoS One. 2025 Jun 24;20(6):e0325547. doi: 10.1371/journal.pone.0325547 (PMC12186958; doi:10.1371/journal.pone.0325547)
Supplement: S5 Table — (DOCX) [file pone.0325547.s005.docx]

**Supplementary Information for “Ediacaran-Cambrian paleosols of Nevada and California” Gregory J. Retallack***, Department of Earth Sciences, University of Oregon. Eugene, Oregon, 97403.*

**Table S5**: **Potassium and boron analyses, Weaver Index (WI) of illite crystallinity, marine-threshold distance (Δ_WI_), ratio of illite/quartz (10Å/2.46Å peaks), XRD-predicted clay (%), and field lithology for selected fossils of southern California and Namibia**.

| Ma | Locality | Formation | Taxon | Condon spem. no. | K % | B ppm | B/K μg/g | WI | Δ_WI_ | Clay/qtz | Clay % | Lithology |
| --- | --- | --- | --- | --- | --- | --- | --- | --- | --- | --- | --- | --- |
| 510 | Cadiz, California | Latham Shale | *Olenellus clarki* (T) | F111543 | 2.9 | 20 | 2.0 | 3.2 | 0.2 | 1.30 | 33 | gray shale |
| 541 | Swartpunt, Namibia | Spitzkopf Mbr | *Manykodes pedum* (R) | F120826 | 0.5 | 10 | 20.0 | 3.1 | 9 | 1.34 | 33 | gray siltstone |
| 543 | Emigrant P., California | Wood Canyon | *Ernietta plateauensis* (V) | F123791A | 1.3 | 10 | 7.7 | 3.1 | -4 | 1.14 | 32 | gray siltstone |
| 543 | Donna Loy, California | Wood Canyon | *Swartpuntia germsi* (V) | F123788 | 1.9 | <10 | 3.7 | 3.0 | -11 | 1.09 | 32 | gray shale |
| 545 | Mt Dunfee, Nevada | Esmeralda Mbr | *Elainabella deepspringensis* (L) | F123797A | 1.6 | 10 | 6.3 | 3.4 | 4 | 2.74 | 41 | gray shale |
| 545 | Mt Dunfee, Nevada | Esmeralda Mbr | *Conotubus hemiannulatus* (W) | F123798 | 3.6 | 10 | 2.8 | 3.4 | 1.1 | 1.13 | 32 | gray shale |
| 546 | Swartpunt, Namibia | Feldshuhorn M. | *Pteridinium simplex* (V) | F120823B | 1.7 | <10 | 4.1 | 2.9 | -12 | 1.58 | 34 | green siltstone |
| 546 | Swartpunt, Namibia | Feldshuhorn M. | *Ernietta plateauensis* (V) | F120825B | 2.4 | <10 | 2.9 | 2.9 | -15 | 2.74 | 41 | green siltstone |
| 548 | Ernietta Hill, Namib. | Aar Member | *Rangea scheiderhoehni* (V) | F120812 | 0.5 | <10 | 14.0 | 2.8 | -6 | 1.11 | 32 | gray siltstone |
| 549 | Aarhausen, Namibia | Mooifontein M. | *Beltanelliformis brunsae* (D) | F120803 | 0.3 | <10 | 23.3 | 2.9 | 6 | 1.12 | 32 | gray siltstone |
| 550 | Pockenbank, Namib. | Kanies Member | *Ernietta plateauensis* (V) | F120819 | 3.6 | <10 | 1.9 | 2.7 | -22 | 1.26 | 33 | red siltstone |
| 565 | Donna Loy, California | Stirling Quartz. | *Hallidaya brueri* (V) | F123782A | 3.8 | 10 | 3.7 | 3.1 | -10 | 1.75 | 35 | gray siltstone |
| 580 | Donna Loy, California | Johnnie Form. | *Boxonia pertaknurra* (O) | F123779A | 0.6 | 10 | 16.7 | 3.3 | 11 | 1.52 | 34 | limestone |

Codes in taxon column: (D) discoid microbial colony, (L) algae, (O) stromatolite, (R) trace fossil, (S) non-crystic paleosol, (T) trilobite, (V) vendobiont, (W) worm. All specimens in Condon Collection, University of Oregon (online catalog paleo.uoregon.edu).
