# Supplementary material for: Ediacaran-Cambrian paleosols of Nevada and California
Source: PLoS One. 2025 Jun 24;20(6):e0325547. doi: 10.1371/journal.pone.0325547 (PMC12186958; doi:10.1371/journal.pone.0325547)
Supplement: S6 Table — (DOCX) [file pone.0325547.s006.docx]

**Supplementary Information for “Ediacaran-Cambrian paleosols of Nevada and California” Gregory J. Retallack***, Department of Earth Sciences, University of Oregon. Eugene, Oregon, 97403.*

**Table S6.** **Interpretation of Bk metrics for Ediacaran and Cambrian paleosols of California**

| Location | Coordinates (^o^N.W) | Lev-el (m) | Age (Ma) | Depth to Bk or By (cm) | Thick-ness Bk or By (cm) | Nod-ule size (cm) | Burial depth (km) | MAP (mm) | MARP (mm) | Time (kyr) | | Soil CO_2_ (ppm) |
| --- | --- | --- | --- | --- | --- | --- | --- | --- | --- | --- | --- | --- |
|  |  |  |  |  |  |  |  |  |  |  |  | |
| Emigrant P. | 25.89018,116.07684 | 105.6 | 512.7 | 26 | 25 | 4 | 4.006 | 403 | 48 | 11.0 | | 1739 |
| Emigrant P. | 25.89018,116.07684 | 102.1 | 512.8 | 45 | 40 | 2 | 4.010 | 563 | 69 | 11.0 | | 2580 |
| Emigrant P. | 25.89018,116.07684 | 99.4 | 512.8 | 35 | 15 | 2 | 4.013 | 482 | 34 | 11.0 | | 2138 |
| Emigrant P. | 25.89018,116.07684 | 85.9 | 513.1 | 55 | 20 | 3 | 4.026 | 636 | 41 | 16.5 | | 3025 |
| Emigrant P. | 25.89018,116.07684 | 84.7 | 513.1 | 41 | 10 | 2 | 4.027 | 532 | 27 | 11.0 | | 2404 |
| Emigrant P. | 25.89018,116.07684 | 75.4 | 513.3 | 40 | 20 | 3 | 4.037 | 524 | 41 | 16.5 | | 2361 |
| Emigrant P. | 25.89018,116.07684 | 74.6 | 513.3 | 42 | 25 | 3 | 4.037 | 540 | 48 | 16.5 | | 2450 |
| Emigrant P. | 25.89018,116.07684 | 73.3 | 513.3 | 30 | 20 | 1 | 4.039 | 439 | 41 | 5.5 | | 1918 |
| Donna Loy | 35.81237,116.08010 | 46 | 569.1 | 14 | 12 | 2 | 4.112 | 288 |  | 9.1 | | 1451 |
| Donna Loy | 35.81237,116.08010 | 15 | 584.9 | 12 | 10 | 2 | 4.112 | 267 |  | 10.0 | | 1300 |

*Note:Duration (Time kyr) of gypsic paleosols (Donna Loy) estimated from 12 and 16% cover, but age of calcic paleosols (Emigrant Pass) from nodule size*
