# Supplementary material for: Ediacaran-Cambrian paleosols of Nevada and California
Source: PLoS One. 2025 Jun 24;20(6):e0325547. doi: 10.1371/journal.pone.0325547 (PMC12186958; doi:10.1371/journal.pone.0325547)
Supplement: S7 Table — (DOCX) [file pone.0325547.s007.docx]

**Supplementary Information for “Ediacaran-Cambrian paleosols of Nevada and California” Gregory J. Retallack***, Department of Earth Sciences, University of Oregon. Eugene, Oregon, 97403.*

**Table S7.** **Paleoclimate and phosphorus depletion inferred from chemical composition of Ediacaran-Cambrian paleosols, California**

| Location | | Pedotype | Coordinates (^o^N.W) | | | | Level (m) | | Age (Ma) | | MAT ^o^C -AI | | MAT ^o^C -CIW | CIA (%) | Tau P (mole fraction) | |
| --- | --- | --- | --- | --- | --- | --- | --- | --- | --- | --- | --- | --- | --- | --- | --- | --- |
|  | |  |  | | | |  | |  | |  | |  |  |  | |
| Cadiz | | Buinga | 34.53564,115.47716 | | | | 19 | | 513.1 | | 13.5 | | 7.2 | 67 | -0.27 | |
| Cadiz | | Bisapi | 34.53564,115.47716 | | | | 7.5 | | 513.4 | | 11.5 | | 8.9 | 68 | -0.13 | |
| Emigrant Pass | | Bui | 35.889276, 116.076449 | | | | 73.2 | | 513.3 | | 9.9 | | 9.7 | 66 | 0.07 | |
| Emigrant Pass | | Aingebite | 35.889276, 116.076449 | | | | 72.8 | | 513.3 | | 10.0 | | 10.2 | 68 | -0.23 | |
| Emigrant Pass | | Pohonta | 35.889276, 116.076449 | | | | 72.3 | | 513.4 | | 9.9 | | 9.2 | 65 | -0.07 | |
| Donna Loy | | Hebinga | 35.812373, 116.080104 | | | | 15 | | 584.9 | | 4.7 | | 10.4 | 49 | -0.55 | |
|  |  | | |  |  |  | |  | |  | |  | |  | |  |
